# Targeting drug resistance in breast cancer: a dual-perspective landscape analysis of PROTACs from bench to bedside

**DOI:** 10.1186/s13058-026-02320-w

**Published:** 2026-06-09

**Authors:** Yinlong Yang, Yihao Lin

**Affiliations:** 1 Department of Breast Surgery, Fudan University Shanghai Cancer Center, Shanghai, 200032 China; 2 Department of Oncology, Shanghai Medical College, Fudan University, Shanghai, 200032 China; 3 Department of General Surgery, Ruijin Hospital, Shanghai Jiao Tong University School of Medicine, Shanghai, China

**Keywords:** Proteolysis targeting chimeras, Breast cancer, Clinical trials, Bibliometric, Drug resistance

## Abstract

**Background:**

Acquired therapy resistance, particularly endocrine resistance in estrogen receptor-positive (ER +) breast cancer, remains a major clinical challenge. Proteolysis-targeting chimeras (PROTACs) have emerged as a novel therapeutic paradigm with the potential to overcome resistance by degrading target proteins. This study provides a comprehensive landscape analysis of PROTACs in breast cancer, synthesizing research trends and clinical trial progress to map the path from discovery to application.

**Methods:**

We conducted a dual-perspective analysis. Bibliometric data from 350 publications (Web of Science Core Collection) were analyzed to delineate the research evolution, collaboration networks, and target focus. Concurrently, 30 clinical trials registered on ClinicalTrials.gov were systematically reviewed to evaluate the clinical translational stage, target distribution, and safety profiles of PROTACs in breast cancer.

**Results:**

Research output has grown exponentially since 2016, led by China and the United States. The estrogen receptor (ER) is the predominant target in both basic and clinical research. Clinical development is advancing rapidly, with the ER degrader ARV-471 reaching phase III trials. In patients with ER + /HER2 − advanced breast cancer and ESR1 mutations, ARV-471 demonstrated a median progression-free survival of 5.0 months, doubling that achieved with fulvestrant (2.1 months). While fatigue and nausea are common adverse events, the overall safety profile appears manageable. However, clinical exploration remains highly concentrated on ER + disease, with limited progress in other subtypes such as HER2 + and triple-negative breast cancer.

**Conclusions:**

PROTACs, especially ER degraders, suggest a potential strategy for addressing endocrine resistance in advanced ER + breast cancer, as reflected in encouraging preliminary clinical data. This integrated analysis highlights the rapid translation of this technology while underscoring the critical need to expand target scope, address inherent resistance mechanisms, and broaden applications to other breast cancer subtypes. Our article synthesizes current knowledge and trial landscape, thereby providing a consolidated foundation to inform future research and clinical development of PROTACs in breast cancer.

**Supplementary Information:**

The online version contains supplementary material available at 10.1186/s13058-026-02320-w.

## Introduction

Breast cancer is one of the most common malignant tumors [1]. Traditional small-molecule drugs commonly used in breast cancer treatment primarily target the direct functional binding sites or allosteric sites of proteins [2]. Although such therapies have significantly improved the survival outcomes of breast cancer patients, acquired resistance remains one of the primary causes of treatment failure [3]. Such resistance severely limits the long-term efficacy and clinical benefits of traditional therapies [4]. Consequently, developing novel therapeutic strategies capable of targeting “undruggable” proteins to more comprehensively block oncogenic signaling pathways has become a critical direction in breast cancer research.

Proteolysis-targeting chimeras (PROTACs) are among the most advanced targeted protein degradation technologies to date [5]. PROTACs can induce the degradation of specific proteins of interest (POI) and PROTACs consist of three components: an E3 ubiquitin ligase, a POI ligand, and a linker [6]. E3 ubiquitin ligase is used for the specific selection of substrates for ubiquitination and degradation. The POI ligand acts as a molecule to hijack the POI, and the linker can conjugate these two ligands. Compared to traditional inhibitors, PROTACs degrade rather than inhibit target proteins [7]. Beyond their ability to target “undruggable” proteins, PROTACs have demonstrated the capacity to mitigate drug resistance driven by mutations or overexpression [8]. This suggests a promising mechanism for overcoming traditional occupancy-based therapy resistance, though its success is often dependent on factors such as the specific target-ligand interactions and the cellular environment. Besides, it is essential to distinguish between the ability of PROTACs to bypass existing resistance to inhibitors and their own susceptibility to novel, technology-specific resistance mechanisms, such as E3 ligase alterations.

Bibliometrics applies mathematical and statistical methods to analyze the patterns of literature production, citation networks, and co-occurrence frequencies [9]. It can reveal disciplinary development, research hotspots, and academic influence. By identifying emerging frontiers, visualizing knowledge structures, and monitoring the intensity of interdisciplinary integration, bibliometrics has become a key tool for scientific research and data analysis [10].

This study focuses on breast cancer and systematically analyzes the developmental trajectory of PROTACs in breast cancer from dual perspectives of bibliometrics and clinical trials. PROTAC technology has advanced from basic research to clinical development. Targeting the challenge of endocrine therapy resistance in breast cancer, ER has become a key focus for development. As a representative drug, ARV-471 has entered Phase III clinical trials. However, PROTACs themselves still face potential resistance risks, such as E3 ligase downregulation and target protein mutations.

## Materials and methods

### Data sources and search strategy

Scientific publications were retrieved from the Web of Science Core Collection (WoSCC) database on January 1, 2026. WoSCC is a commonly used database in bibliometric research, offering comprehensive global coverage of academic journal literature. To minimize potential retrieval bias, the search was completed within a single day. The search strategy employed was: (TS = (protac OR protacs OR proteolysis-targeting chimeras OR proteolysis targeting chimera OR proteolysis-targeting chimera OR proteolysis targeting chimeras OR protac degrader OR protac degraders) AND TS = (breast cancer OR breast tumors)). Publication types were restricted to journal articles and reviews. Only English-language publications were included. This search yielded 350 relevant articles published between 2003 and 2025.

**Fig. 1:**
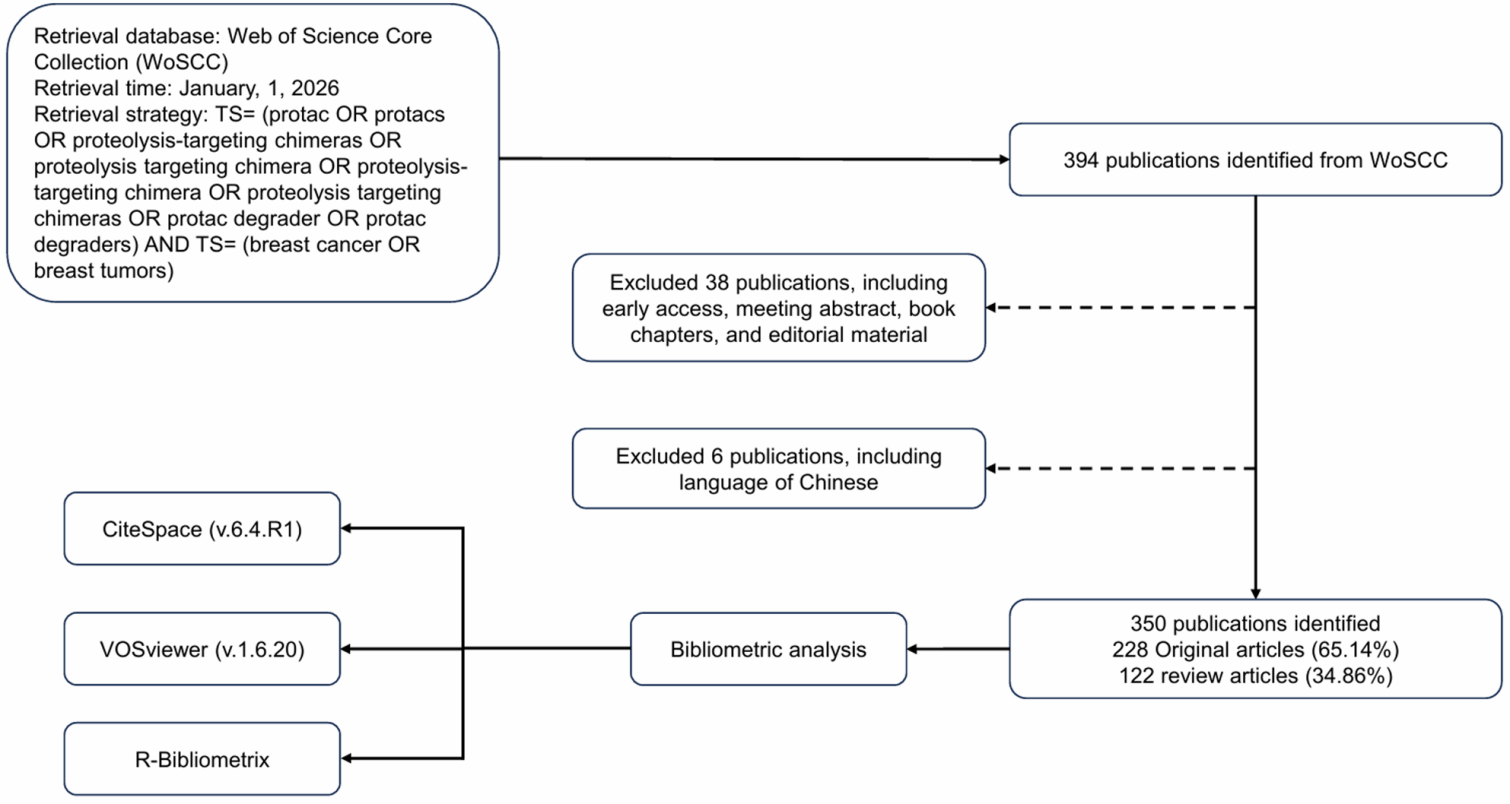
Flowchart of the search strategy and exclusion criteria

To ensure the accuracy and interpretability of the bibliometric visualization, a systematic keyword normalization process was conducted using the thesaurus function in VOSviewer and the alias list in CiteSpace. Synonymous terms and spelling variations—including “PROTAC,” “PROTACs,” “proteolysis-targeting chimeras,” and “targeted protein degradation”—were merged into a single representative node to prevent data redundancy. Similarly, biological targets with multiple designations, such as “ER” and “estrogen receptor”, were harmonized into a standardized format. This cleaning step allowed for a more precise identification of research hotspots and evolution trends within the breast cancer PROTACs landscape. Finally, full records and cited references were exported in plain text format, and the literature search process is documented in Fig. 1.

### Bibliometric analysis

Bibliometrics serves as a methodology that employs visualization software to conduct systematic analysis of topic-related literature, thereby exploring research hotspots and forecasting future trends within relevant academic domains. We used the CiteSpace (v.6.4.R1), VOSviewer (v.1.6.20), and R-Bibliometrix visualization software for analysis. The time span was set to be from January 2003 to December 2025 and the time slice was one year. In the study, the parameter Top N = 50 was set to filter out the 50 items with the highest frequency in each time slice. The threshold of Top N = 50 was chosen to balance network clarity and data representativeness. Sensitivity analyses conducted at N = 25 and 100 yielded qualitatively consistent results, confirming that the core research clusters and trends are robust and not sensitive to variations in this filtering parameter.

### Clinical trial analysis

Clinical trial data were retrieved from the authoritative global clinical trial registry platform ClinicalTrials.gov on January 1, 2026. To ensure a comprehensive and reproducible landscape analysis and minimize potential retrieval bias, the search was completed within a single day. The search logic was defined as follows: Condition/Disease: “Breast Cancer” OR “Breast Neoplasms” OR “Breast Tumors”; Intervention/Treatment: “PROTAC” OR “Proteolysis Targeting Chimera” OR “Targeted Protein Degradation” OR “Protein Degrader”. This was further supplemented by manual searches for specific drug candidates, including ARV-471, AC682, AC699, HRS-1358, and HP568. Inclusion criteria encompassed trials explicitly utilizing PROTAC technology for breast cancer patients, early-phase solid tumor trials featuring breast cancer-specific expansion cohorts, and studies in healthy volunteers evaluating the pharmacokinetics or safety of these degraders. Conversely, exclusion criteria included non-PROTAC targeted therapies, duplicate trial registrations, and studies lacking breast cancer-relevant information.

To ensure statistical rigor, clinical trial analyses were conducted using a trial-level counting approach (N = 30). For data normalization, sponsors and geographical attributions were defined by the headquarters location of the primary “Lead Sponsor” as registered on ClinicalTrials.gov to prevent redundancy. In cases of co-sponsored or multi-center trials, only the primary lead sponsor was counted as the representative entity to establish the denominator. This methodology ensures that each trial is weighted equally, regardless of the number of individual study sites or secondary collaborators involved. Furthermore, adverse events were systematically extracted from either the registry or linked academic publications. Key parameters (including therapeutic targets, trial phases, and research institutions) were synthesized for the 30 relevant trials identified. This analysis aimed to characterize the current clinical landscape and future developmental trajectories of PROTAC molecules in breast cancer therapeutics.

### Enrichment analysis

This study applied the clusterProfiler package in R statistical programming language to perform Gene Ontology functional annotation and Kyoto Encyclopedia of Genes and Genomes pathway enrichment analysis on target genes. Based on a statistical significance threshold of *P*-value < 0.05, significantly enriched biological function terms and signaling pathways were screened out.

## Results

### Analysis of annual publication

**Fig. 2:**
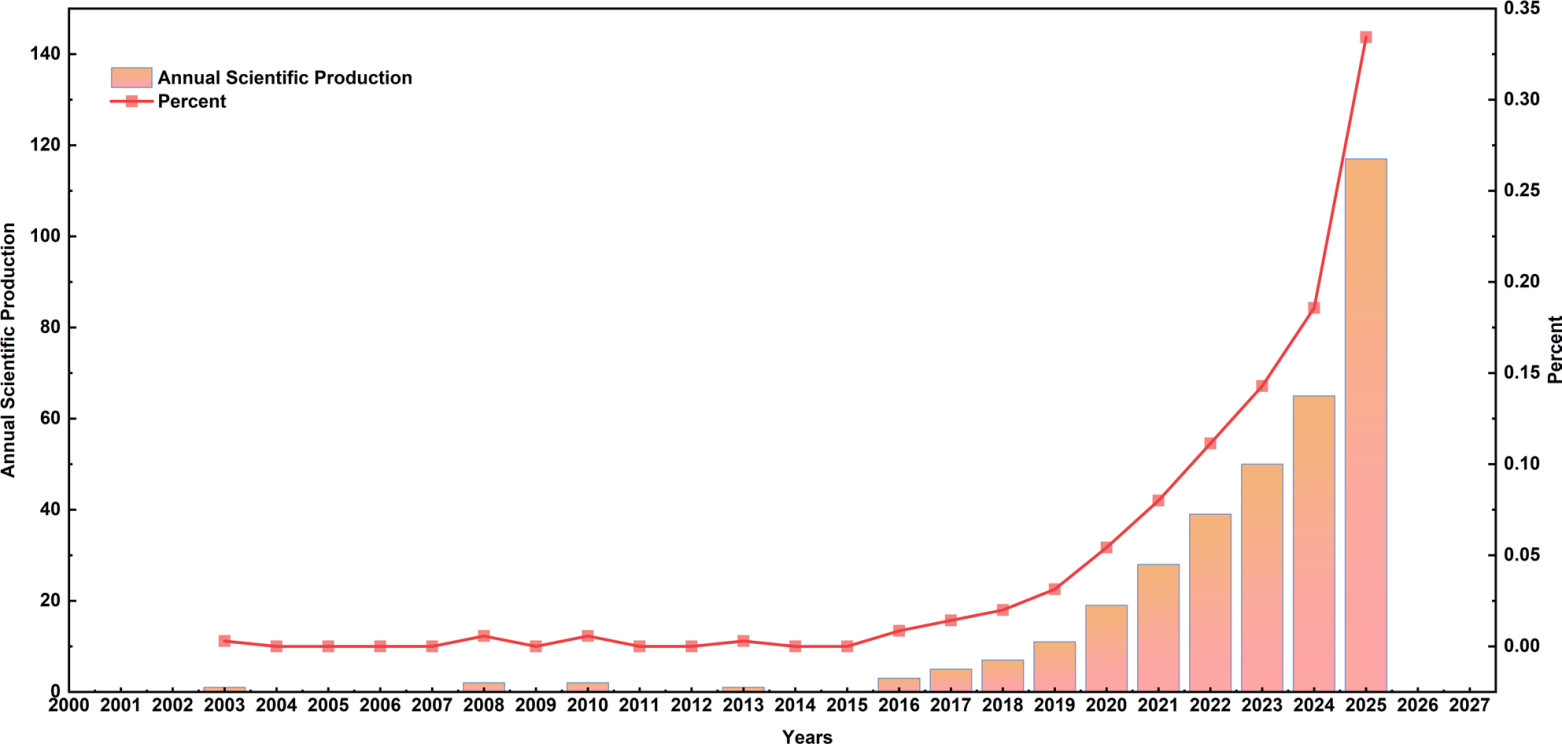
Number and trend of annual publication

This study analyzed a total of 350 publications, including 228 original articles (65.14%) and 122 review articles (34.86%). Study plotted the annual paper output (Fig. 2) and publication trends reveal two distinct phases: (1) Initial development (2003–2015): Following the first publication in 2003, the average annual publication output remained below 5 papers for the subsequent 12 years, with no relevant publications for several consecutive years. This low and fluctuating publication output is characteristic of an emerging field. (2) Rapid expansion (2016-Present): Beginning with 3 publications in 2016, annual output has shown consistent growth, reaching 117 publications in 2025. During this phase, the annual growth rate consistently exceeded 25%, notably reaching a peak of 80% in 2025. Based on the current trends, output is projected to continue increasing. These data demonstrate that the application of PROTACs in breast cancer drug resistance has progressively become a significant research focus.

### Analysis of countries/regions and institutions

**Fig. 3:**
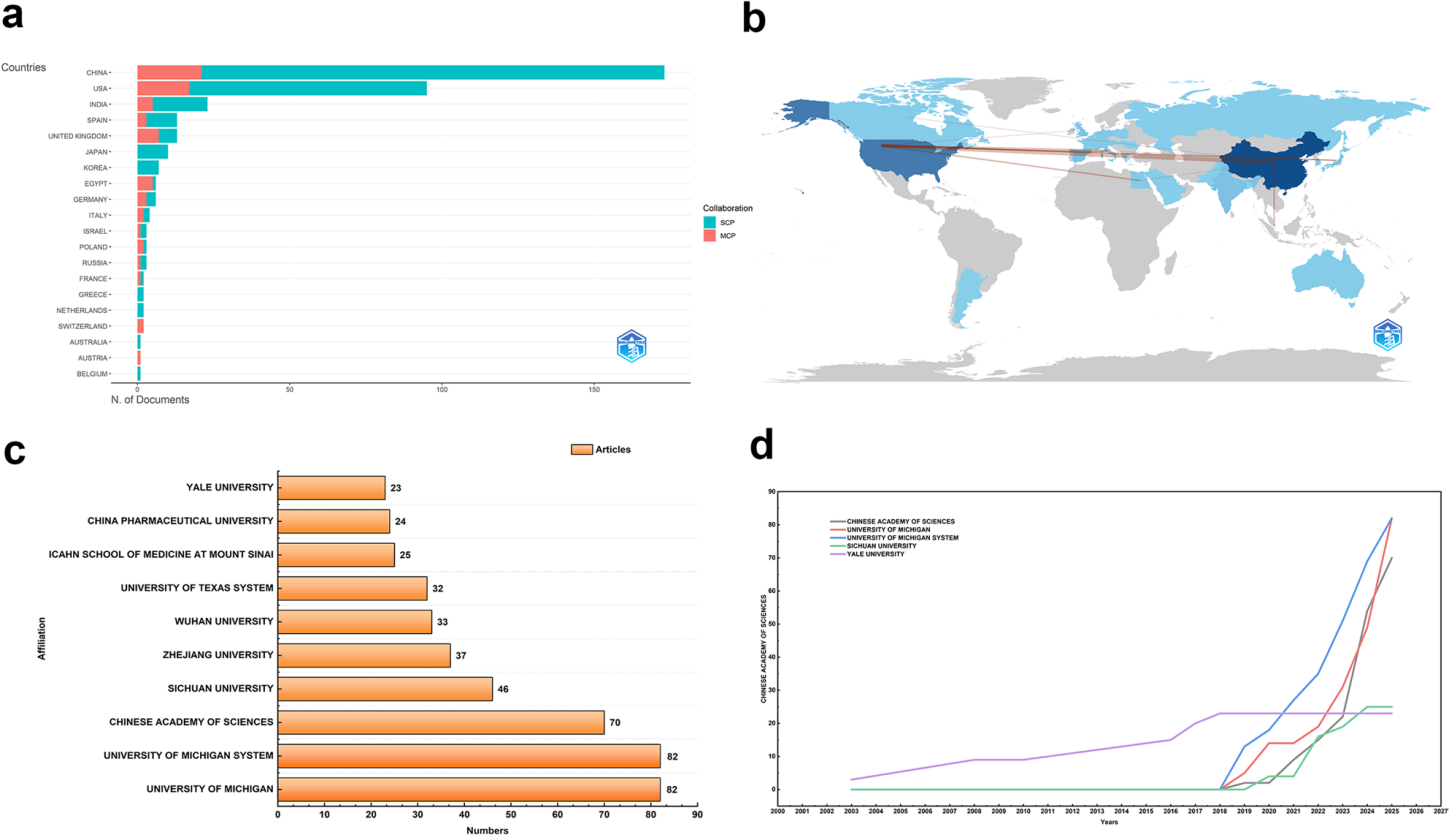
**a** Country distribution of publication output; **b** Country collaboration in publication output; **c** The top 10 institutions with the highest publication output; **d** Yearly publication trends of top institutions

Publications in this study originated from 33 countries/regions and 552 institutions. China (N = 173) and the United States (N = 95) lead in publication volume, indicating their dominant roles in PROTACs and breast cancer research (Fig. 3a). Notably, China and the United States also maintain strong collaborative link (Fig. 3b). In the figure, thicker and darker lines represent stronger research collaborations, while deeper blue shading on the map corresponds to higher national publication counts. This centrality within the global collaboration network underscores their extensive international cooperation in this field.

This study analyzed all 552 contributing institutions and identified the top 10 by publication volume (Fig. 3c). Notably, the top 10 institutions are all distributed between China and the United States. The annual output of publishing institutions is presented in this study (Fig. 3d). These institutions show rapid growth in publications since 2016. This aligns with our previous annual publication analysis, with both indicating sustained and growing research interest in PROTACs for breast cancer applications from 2016 onward.

### Analysis of authors and sources

**Fig. 4:**
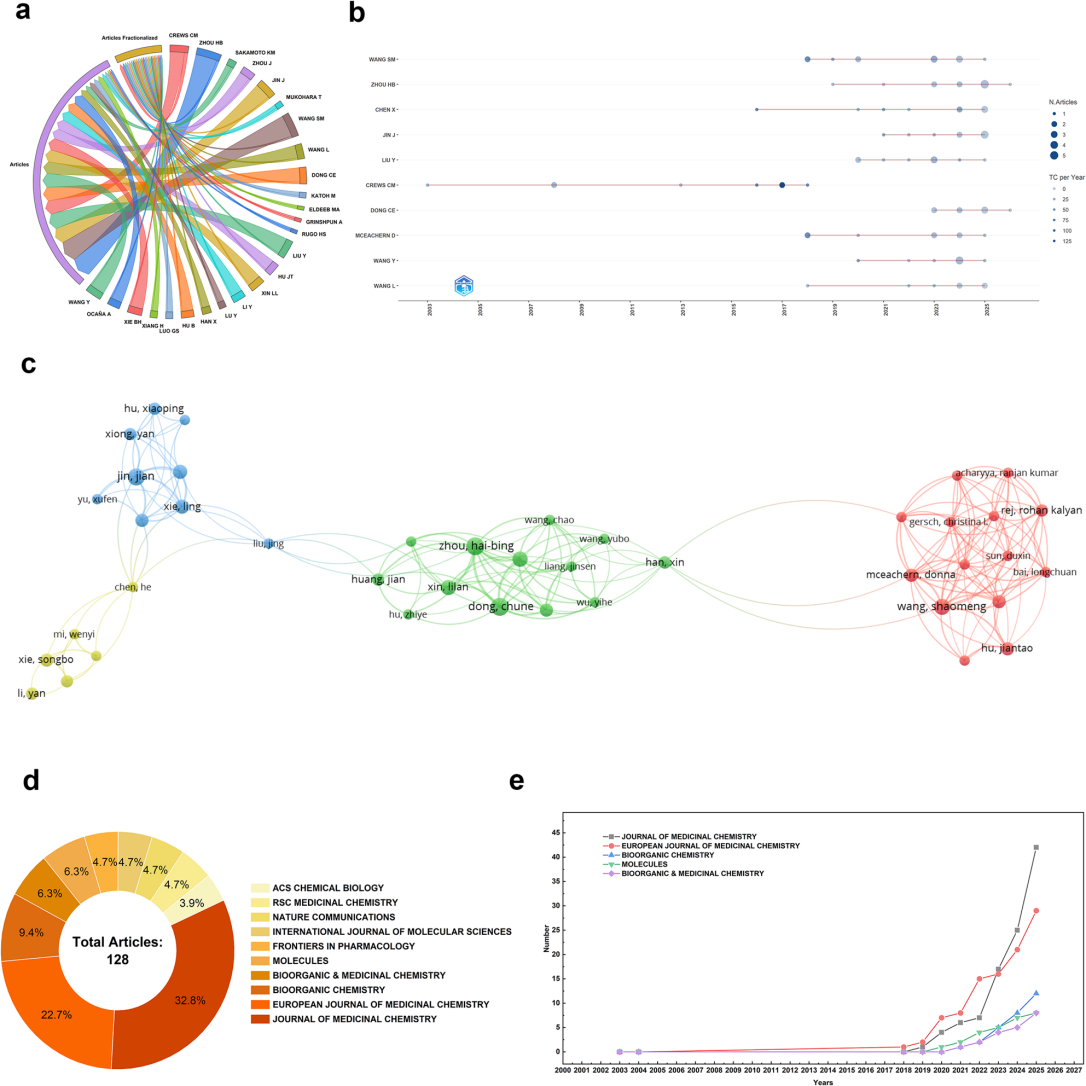
**a** Chord diagram of authors’ publication output; **b** Annual publication output of top authors; **c** Authors co-occurrence network; **d** The top 10 journals with the highest publication output; **e** Yearly publication trends of top journals

A total of 2460 scholars authored 350 publications. The top 25 authors by publication count are visualized in a chord diagram (Fig. 4a). Fractionalized article count, which reflects individual contribution in collaborative work, highlights Craig M. Crews (2.02). Analysis of annual author publication volume reveals Craig M. Crews as the sole publishing author between 2003 and 2015 (Fig. 4b). Combining publication volume and fractional contribution, Crews’ sustained leadership in the field are evident. From 2019 onward, both author numbers and publications surged, indicating a flourishing research landscape. Analysis of the author collaboration network reveals a high-density cooperative network within field, demonstrating strong collaborative relationships among researchers (Fig. 4c).

All publications originated from 166 distinct sources; the top 10 most prolific journals are shown in Fig. 4d. Notably, high-output journals predominantly specialize in chemistry and pharmacology, indicating PROTAC research has progressed from fundamental concept validation to active chemical development. Annual publication trends for the top 5 journals are visualized in Fig. 4e. All five journals show a steady increase in output since 2018. This confirms the rapid development of PROTAC in breast cancer research over the past decade.

### Analysis of keywords and reference

**Fig. 5:**
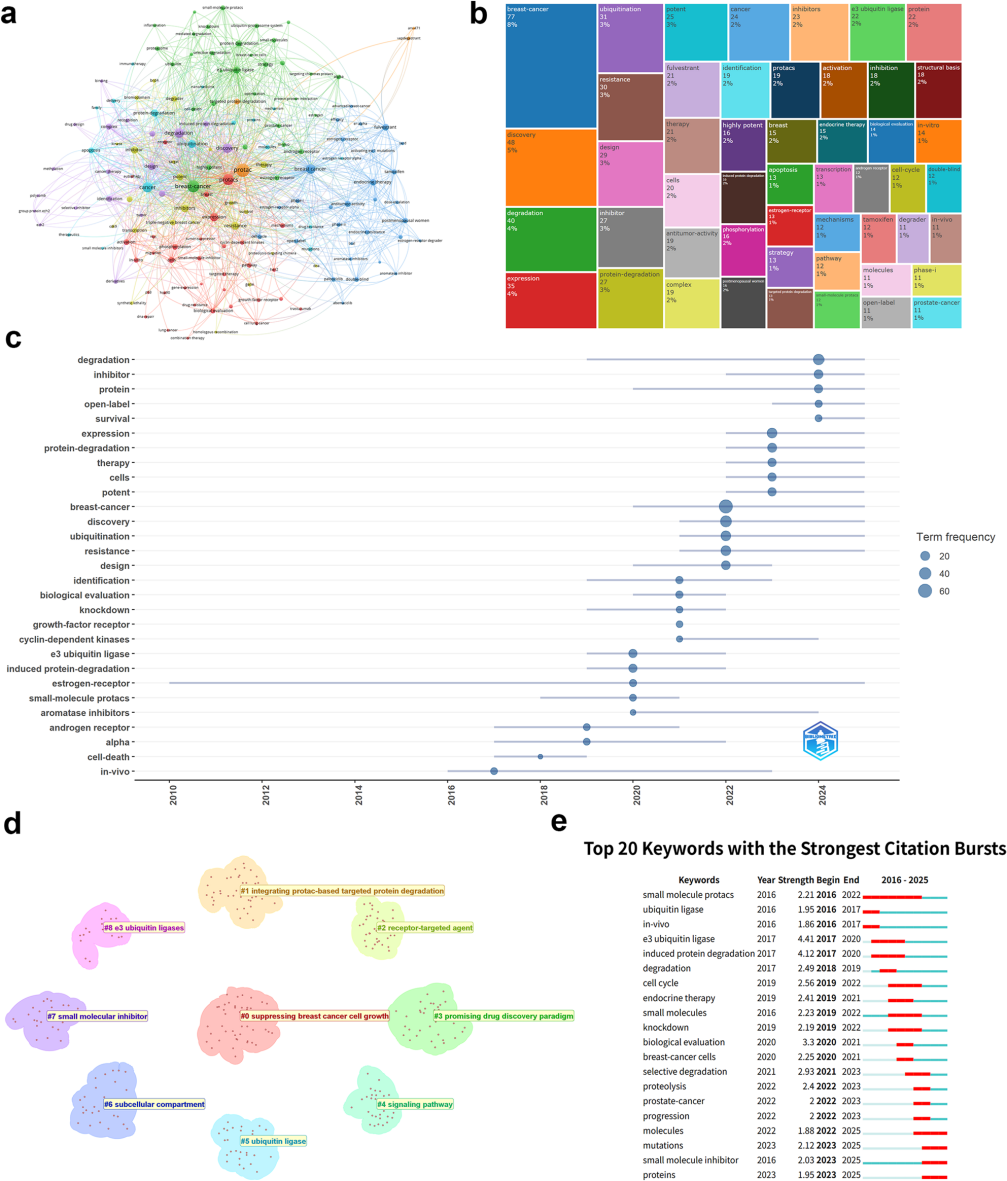
**a** Keywords co-occurrence network; **b** Tree diagram of keywords; **c** Analysis of topic trends in publications; **d** Clustering of keywords; **e** The top 20 keywords with the strongest citation bursts

The study visualized the co-occurrence network of 159 keywords occurring 5 or more times (Fig. 5a). A corresponding tree map of keywords is shown in Fig. 5b. As evidenced by key processes such as discovery (5%), expression (4%), and ubiquitination (3%), PROTAC research in breast cancer comprehensively spans mechanistic investigations to therapeutic efficacy evaluations. Trend topic analysis (Fig. 5c) shows sustained focus on the ER since 2010. Importantly, as a high-frequency keyword and trending topic, resistance is a hot direction in PROTACs research. This indicates the significant value of this technology as a novel approach to overcoming the drug resistance issues associated with traditional therapies. Keyword cluster analysis (Fig. 5d) revealed PROTACs are frequently compared with small molecule inhibitors for breast cancer in research. Notably, such comparative studies highlight the potential of PROTACs as a promising drug discovery paradigm, significantly boosting both the popularity and depth of related research. Keyword burst analysis identified significant surges in attention (Fig. 5e). The rise of “induced protein degradation (2017)” “endocrine therapy (2019)” “breast cancer cells (2020)” and “selective degradation (2021)” demonstrates the three-phase transition of PROTAC technology: from molecular design, to disease validation, and finally to efficacy optimization.

This research summarizes the top 10 most cited publications and presents their annualized citations (Table 1). Among these, four are reviews, highlighting researchers’ heightened demand for understanding the disciplinary knowledge architecture. The highest-cited paper is Sakamoto KM’s 2001 study in *Proceedings of the National Academy of Sciences of the United States of America* [6]. This article reveals how PROTACs serve as a revolutionary strategy by hijacking the SCF E3 ubiquitin ligase complex to achieve precise targeted degradation of specific disease-causing proteins. The article with the highest annualized citations is by Békés M et al., published in *Nature Reviews Drug Discovery* in 2022 [20]. This paper systematically reviews the key breakthroughs during the first two decades of PROTACs technology development, assesses the current landscape, and explores future research priorities.

**Table 1:** The most cited ten publications

Rank	Title	Journal	First author	Citations	Year	Annualized citations
1	Protacs: chimeric molecules that target proteins to the Skp1-Cullin-F box complex for ubiquitination and degradation [6]	Proc Natl Acad Sci USA	Sakamoto KM	96	2001	3.84
2	PROTAC targeted protein degraders: the past is prologue [25]	Nat Rev Drug Discov	Békés M	75	2022	18.75
3	DRUG DEVELOPMENT. Phthalimide conjugation as a strategy for in vivo target protein degradation [26]	Science	Winter GE	60	2015	5.45
4	Catalytic in vivo protein knockdown by small-molecule PROTACs [27]	Nat Chem Biol	Bondeson DP	53	2015	4.82
5	Induced protein degradation: an emerging drug discovery paradigm [28]	Nat Rev Drug Discov	Lai AC	44	2017	4.89
6	Structural basis of PROTAC cooperative recognition for selective protein degradation [29]	Nat Chem Biol	Gadd MS	38	2017	4.22
7	Discovery of ERD-308 as a Highly Potent Proteolysis Targeting Chimera (PROTAC) Degrader of Estrogen Receptor (ER) [30]	J Med Chem	Hu JT	37	2019	5.29
8	Small-Molecule PROTACS: New Approaches to Protein Degradation [31]	Angew Chem Int Ed Engl	Toure M	35	2016	3.50
9	Selective Small Molecule Induced Degradation of the BET Bromodomain Protein BRD4 [32]	ACS Chem Biol	Zengerle M	34	2015	3.09
10	Proteolysis-Targeting Chimeras as Therapeutics and Tools for Biological Discovery [33]	Cell	Burslem GM	32	2020	5.33

### Analysis of targets

**Fig. 6:**
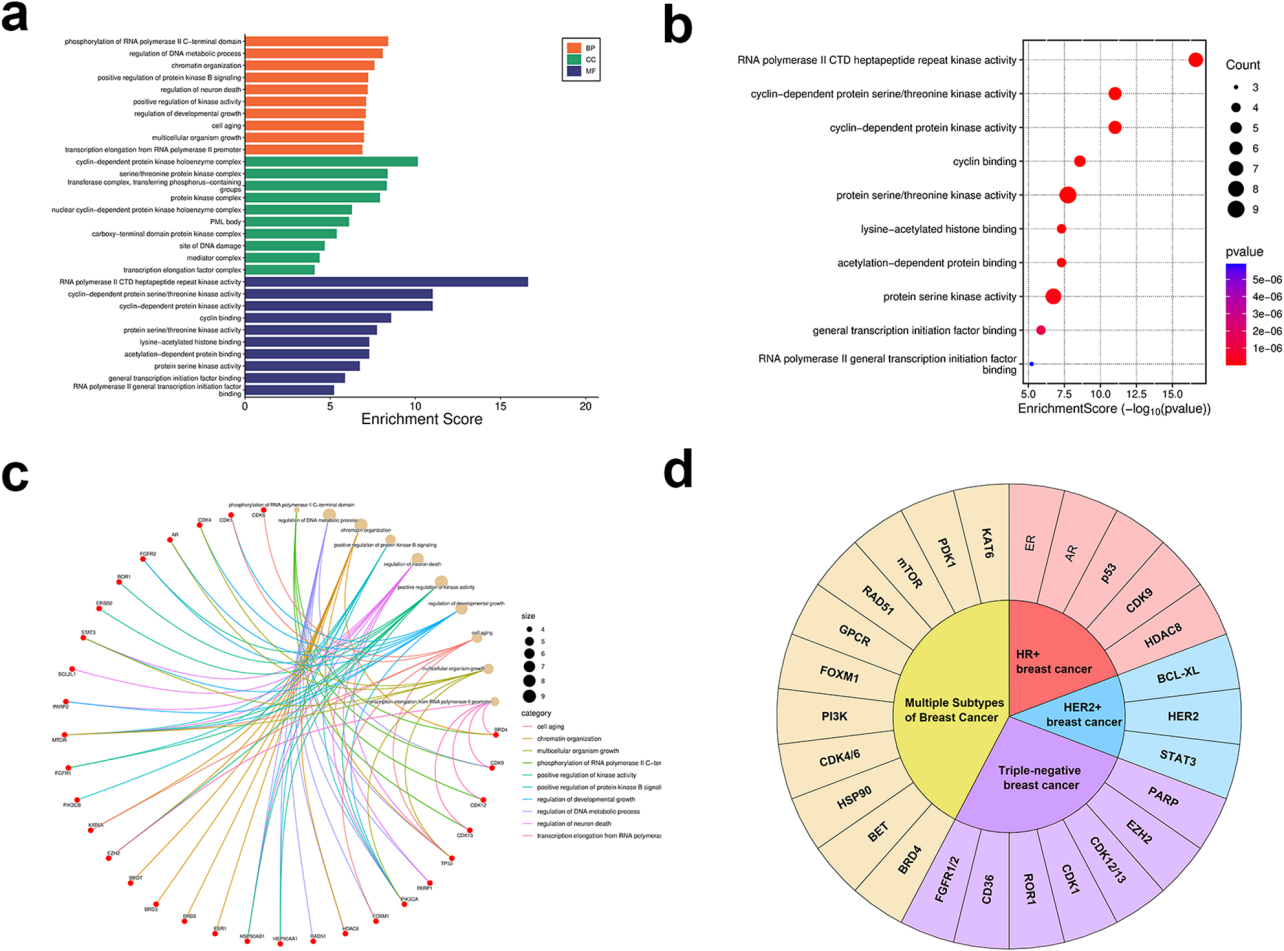
**a** Enrichment analysis of gene three ontologies; **b** Enrichment analysis of molecular function; **c** Analysis of gene interaction networks and functional linkage; **d** Analysis of the relationship between genes and breast cancer subtypes

This study summarized 26 targets involved in the publications, with the ER being the most extensively studied (Table S1). GO enrichment analysis and molecular function analysis of the genes associated with these targets (Fig. 6a, b) revealed that their functions are significantly enriched in signal transduction and transcriptional regulation pathways, with deep involvement in cell cycle regulation. Gene interaction network and functional association analyses further confirmed that these genes widely participate in multiple biological processes, including cellular senescence, DNA repair, and cell cycle regulation (Fig. 6c). Collectively, these analyses suggest that PROTACs in breast cancer research primarily focus on the dual core biological mechanisms of endocrine signaling and cell cycle regulation. Additionally, this study analyzed the distribution of relevant targets across different breast cancer subtypes (Fig. 6d). The results indicate that the targets involved in PROTAC research are widely distributed across multiple breast cancer subtypes, including hormone receptor positive (HR +), human epidermal growth factor receptor 2 positive (HER2 +), and triple-negative breast cancer (TNBC). Comprehensive target analysis indicates that PROTACs research targeting breast cancer drug resistance has covered multiple subtypes and targets, with ER + breast cancer and its related targets ER being the main focus.

### Analysis of PROTACs clinical trials

**Fig. 7:**
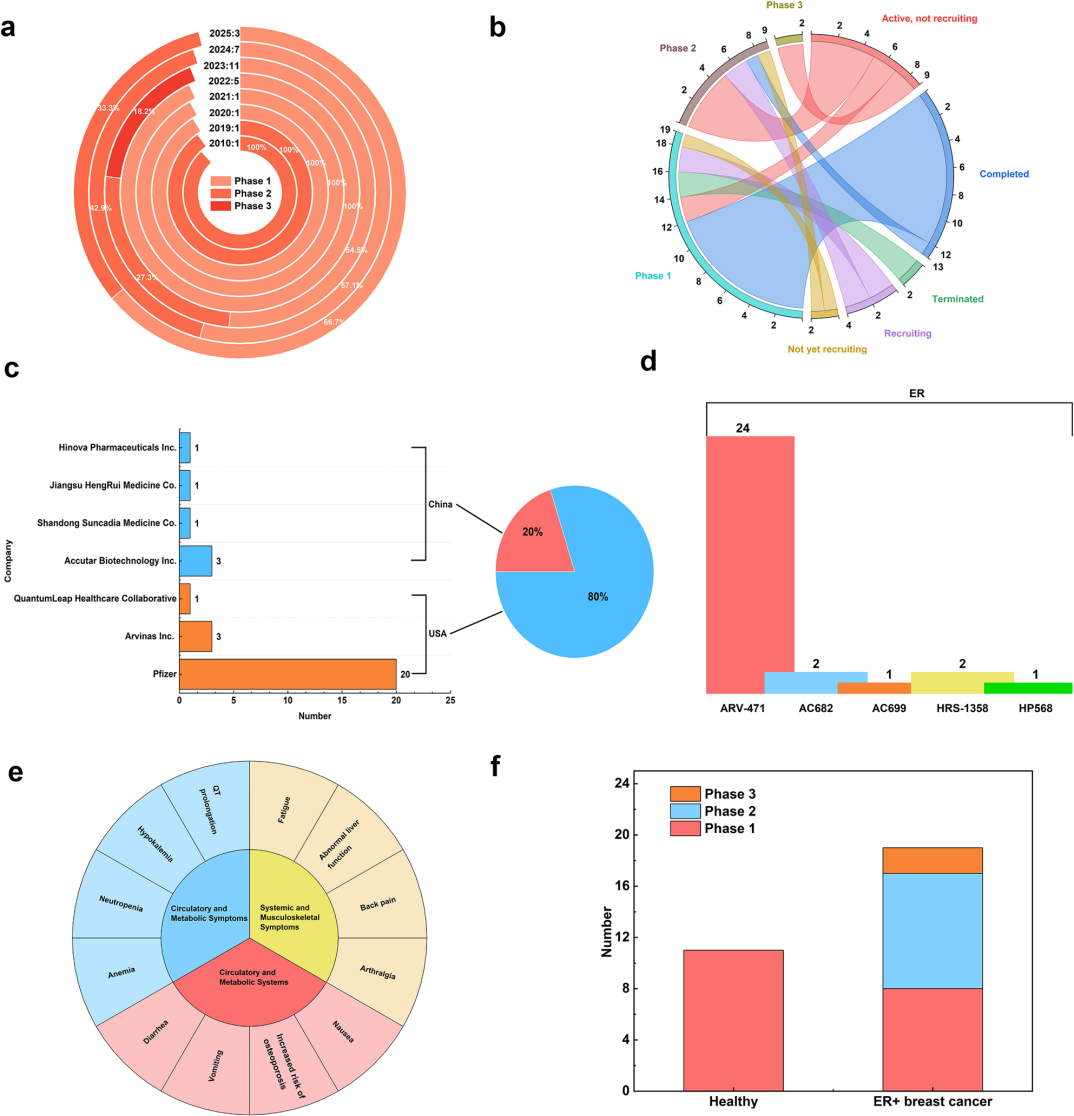
**a** Distribution of clinical trials started year and phase; **b** Distribution of clinical trials phase and status; **c** Distribution of clinical trials sponsor companies; **d** Distribution of clinical trial targets; **e** Summary of adverse events from a published result of the phase III clinical trial of ARV-471 in advanced breast cancer (NCT05654623); **f** Distribution of clinical trial participant subtypes

Preliminary studies revealed that the targets investigated in PROTAC basic research involve multiple breast cancer subtypes. We further analyzed PROTACs clinical trials. Database interrogation identified 30 relevant clinical trials (Table S2). The majority remain in Phase I (n = 19) (Fig. 7a), with two landmark trials progressing to Phase III (NCT05909397, NCT05654623). Notably, the trial (NCT05654623) evaluated ARV-471 (200 mg daily) versus fulvestrant in 624 patients with ER + /HER2 − advanced breast cancer following progression on CDK4/6 inhibitors. Randomization was stratified by ESR1 mutation status and the presence of visceral disease. Published efficacy result showed that in the ESR1-mutant subgroup (n = 270), ARV-471 significantly prolonged median PFS to 5.0 months [95% CI, 3.7–7.4] compared to 2.1 months [95% CI, 1.9–3.5] for fulvestrant (HR, 0.58; 95% CI, 0.43–0.78; *P* < 0.001), representing a 42% reduction in the risk of progression. While no statistically significant difference in PFS was reached in the intent-to-treat population (3.8 vs. 3.6 months; HR, 0.83; *P* = 0.07), these findings underscore the superior efficacy of ARV-471 specifically in ESR1-mutated settings [11]. For patients with ER + , HER2- advanced breast cancer harboring ESR1 mutations, ARV-471 demonstrated superior efficacy in delaying disease progression compared to fulvestrant.

The chord diagram shows that the majority of clinical trials are in “completed” and “active, not recruiting” statuses, demonstrating both a solid research foundation and robust clinical research activity in this field (Fig. 7b). Clinical trials in this domain are led by the US (80%) and China (20%), anchored by Pfizer (66.7%) (Fig. 7c). ER is the core target in clinical trials (Fig. 7d). Among agents targeting ER, development efforts centered predominantly on ARV-471. Figure 7e summarizes the adverse events from a published result of the Phase III clinical trial of ARV-471 in advanced breast cancer. The results indicate that treatment-related adverse reactions primarily involve the circulatory and metabolic, musculoskeletal, and digestive systems. Therefore, clinical application should prioritize the monitoring of hematological indices, cardiac electrophysiology, and liver function [12].

Analysis of clinical trial participant characteristics reveals that PROTAC research is primarily focused on the ER + breast cancer subtype (N = 19) (Fig. 7f). In contrast, clinical research on PROTACs for other breast cancer subtypes (such as HER2 + and TNBC) is relatively limited. While the lack of a single, dominant oncogenic driver akin to the ER is a factor, this gap is more accurately attributed to the complex interplay of target druggability, the specific E3 ligase expression landscape within these subtypes, and the high degree of intra-tumoral heterogeneity. Moreover, the vast majority of clinical trials target patients with advanced breast cancer. This may be closely related to the significant resistance these patients develop to endocrine therapy, among other treatments.

This study analyzed the inclusion criteria and conditions of relevant clinical trials (Table S3). We found that the enrolled patients were mostly ER + /HER2 locally advanced or metastatic breast cancer patients who had progressed after prior endocrine therapy and CDK4/6 inhibitor treatment (resistant population). This indicates that PROTACs research focuses on overcoming drug resistance in advanced breast cancer treatment, particularly against endocrine therapy resistance and CDK4/6 inhibitor resistance. Furthermore, a dedicated clinical trial targeting patients with breast cancer scheduled to receive neoadjuvant therapy has been identified. This contrasts with the majority of trials focused on locally advanced or metastatic disease, underscoring the expanding therapeutic scope of PROTACs.

## Discussion

This study offers an integrated analysis of the dual perspectives of bibliometrics and clinical trial analysis, systematically revealing the research landscape and developmental trajectory of PROTACs in the field of breast cancer. The findings reveal a surge in publication volume in this domain in recent years, with this highly active research momentum projected to persist. This suggests that PROTACs have emerged as a core frontier in breast cancer therapeutic research. The ER is currently the predominant target for PROTAC application in breast cancer research. Consequently, related clinical trials are highly concentrated on patient populations with ER + advanced or metastatic breast cancer. Notably, clinical trials for ARV-471, a representative PROTAC drug, have advanced to phase III. This significant breakthrough also provides a reference pathway for subsequent trials to enter phase III. To contextualize this translational momentum, we compared developmental timelines: Palbociclib (CDK4/6 inhibitor) and Elacestrant (oral SERD) took 14 and 17 years, respectively, from discovery to approval [13, 14]. In contrast, ARV-471 progressed from clinical entry in 2019 to positive results in 2025—a mere 6-year trajectory. This acceleration suggests the “degradation paradigm” may outpace traditional small-molecule development, likely due to pre-validated targets like ER and high catalytic efficiency, which facilitate rapid detection of therapeutic efficacy. Research on PROTACs overcoming traditional breast cancer drug resistance is progressing from fundamental molecular design towards validation in disease models, ultimately aiming to optimize clinical therapeutic outcomes.

ER is the central target in breast cancer PROTAC development, driven primarily by the large population of ER + patients and the critical challenge of endocrine therapy resistance. For the highly targeted ER, traditional endocrine therapies (such as tamoxifen and fulvestrant) primarily function by inhibiting ER activity or reducing ligand levels [15]. This makes endocrine therapy susceptible to resistance due to ER gene mutations (e.g., mutations in the ligand-binding domain), ER overexpression, and activation of alternative signaling pathways [16]. In contrast, the degradation of ER by PROTACs not only abolishes its classical genomic transcriptional functions but also eliminates its non-genomic signaling functions, thereby more comprehensively blocking the ER pathway [16]. To date, the trial results have demonstrated that ARV-471 offers a clinically meaningful benefit for pre-treated patients, specifically those harboring ESR1 mutations [11]. In this subgroup, ARV-471 more than doubled the median PFS compared to fulvestrant (HR, 0.58; 95% CI, 0.43–0.78; *P* < 0.001), validating its superior efficacy in overcoming ESR1-mediated endocrine therapy resistance. The lack of statistical significance in the overall ITT population (HR, 0.83; *P* = 0.07) underscores the critical importance of biomarker-driven therapy selection, highlighting that PROTAC-mediated degradation may be most potent in tumors with a high dependency on mutated ER signaling. These findings provide the clinical rationale for the potential regulatory approval of ARV-471 specifically for patients with ESR1-mutant advanced breast cancer.

Building on the successful paradigm of ER-targeting degradation, PROTACs have the potential to extend to broader drug resistance settings. In the field of targeted therapy resistance, HER2-targeting PROTACs can be designed to overcome resistance to HER2 antibodies. For chemotherapy resistance associated with DNA repair resistance, PARP-targeting PROTACs hold promise for treating PARP inhibitor-resistant TNBC patients. Regarding immunotherapy resistance, degrading targets like EZH2 may help remodel the immunosuppressive microenvironment. These scenarios illustrate potential future applications of PROTACs in addressing drug resistance. However, clinical progress in HER2 + and TNBC subtypes has remained relatively protracted, reflecting multidimensional challenges that extend beyond the mere absence of a single dominant driver. From a druggability perspective, HER2-targeting PROTACs must substantiate their unique catalytic superiority and therapeutic differentiation over incumbent ADC and TKI therapies. Regarding the E3 ligase landscape, differential expression levels of ligases such as CRBN or VHL across various subtypes can lead to inconsistent degradation kinetics [17]. The mechanistic feasibility of PROTACs is intrinsically linked to the endogenous expression landscape of E3 ligases within the breast cancer proteome [18]. For instance, the clinical prioritization of CRBN-recruiting degraders in ER + disease capitalizes on the robust expression of CRBN in these tissues, ensuring efficient ternary complex formation [19]. Conversely, variations in VHL expression across other subtypes necessitate the strategic selection of ligands to optimize degradation kinetics. Finally, the high degree of intra-tumoral clonal heterogeneity, particularly prevalent in TNBC, results in a diverse landscape of co-existing signaling dependencies [20]. Consequently, the degradation of a single target protein may only eradicate specific susceptible subclones, potentially facilitating the enrichment and expansion of resistant populations. The apparent contradiction between PROTACs’ ability to overcome resistance and their own susceptibility to it reflects a shift in resistance landscapes. While PROTACs effectively circumvent resistance driven by target protein overexpression or ER mutations that hinder traditional inhibitors, they remain vulnerable to mechanism-dependent resistance. Unlike traditional drugs, PROTAC efficacy relies on a multicomponent system [21]. Thus, resistance can emerge not just from the target itself, but from the functional loss of E3 ligase recruitment or dysregulation of the ubiquitin–proteasome system.

While PROTACs show promising potential to combat multiple types of treatment resistance, these molecules aren’t immune to resistance development themselves. The potential resistance mechanisms of PROTACs include the following aspects: (1) POI-related resistance: mutations occur at the POI binding site, reducing the binding affinity of PROTACs. In addition, tumor cells regulate the expression level of the target protein, causing it to exceed the effective degradation capacity of PROTACs. (2) E3 ligase-related resistance: mutations or downregulation of the E3 ligase affect the binding of PROTACs to the E3 ligase or its own activity. (3) Dysfunction of the ubiquitin–proteasome system: tumor cells may interfere with the ubiquitination and subsequent degradation of target proteins by inhibiting proteasome activity or upregulating deubiquitinase activity. Instead of proteasome inhibitors, future directions include switching to alternative E3 ligases to bypass ligase-specific mutations or downregulation. Furthermore, the development of dual-E3 PROTACs (Homo-PROTACs) that are bifunctional degraders designed to degrade E3 ligases themselves via autoubiquitination may enhance degradation robustness and reduce the likelihood of resistance [22]. Additionally, conditional PROTACs that trigger protein degradation in specific cell types and at specific time points is also an important approach to overcome their own resistance [23].

Currently, the vast majority of PROTACs clinical trials target patients with advanced breast cancer. If PROTACs demonstrate significant efficacy and favorable long-term safety profiles in advanced-stage treatment, their potential application in early-stage breast cancer or neoadjuvant settings could be further explored in the future. To achieve the successful implementation of PROTACs in clinical pathways, a comprehensive understanding and proactive management of their safety profile must be ensured. The vast majority of PROTAC-related adverse events are mild to moderate. The most common side effects of PROTACs include fatigue, elevated transaminases, and nausea [11]. However, severe adverse events such as thrombocytopenia also exist, representing a class-specific toxicity rather than a universal feature of all PROTACs. Studies have found that this may be related to the “off-target” degradation of transcription factors IKZF1 and IKZF3 mediated by CRBN ligands (such as thalidomide derivatives), which can lead to bone marrow suppression [24]. In contrast, this specific hematological toxicity profile may not be observed in PROTACs utilizing alternative E3 ligases, such as VHL, highlighting that safety profiles are highly dependent on the choice of the E3 ligase recruiter.

This study possesses the following advantages: (1) Integrated dual-perspective analysis: This study systematically combines bibliometric analysis to reveal macro trends with clinical trial data to reflect the current translational landscape and bottlenecks. (2) Precise focus on breast cancer: This study provides the panoramic quantitative analysis of PROTACs spanning from basic research to clinical applications in specific cancer types, offering a unique resource for researchers, drug developers, clinicians, and investors. (3) Multidimensional analysis: We analyze not only the phase distribution, target molecules, and sponsoring companies but also summarize observed and potential adverse reactions.

The findings of this study should be interpreted within its methodological framework, where bibliometric trends and clinical trial registries serve as indicators of scholarly focus and developmental momentum rather than direct therapeutic validation. While the progression of candidates like ARV-471 to Phase III is a notable development, the currently available findings are best characterized as preliminary clinical data, which remain to be validated by definitive confirmatory evidence. Ultimately, the established clinical utility of PROTACs in overcoming endocrine resistance remains contingent upon the results of ongoing randomized controlled trials; thus, our conclusions underscore field-wide research priorities and potential rather than proven clinical efficacy.

The study also has the following noteworthy limitations: (1) Limitations of data sources: The bibliometric analysis relies solely on the WoSCC database, which may result in incomplete coverage of regional or discipline-specific research outputs, thereby affecting the comprehensiveness of the knowledge map; the analysis of clinical trials depends entirely on the ClinicalTrials.gov registry platform, which may not include all relevant studies, particularly ongoing or unpublished early-phase trials. (2) Limitations of language inclusion:​ The study’s inclusion criteria were restricted to English-language publications. This language restriction may have led to the exclusion of relevant research published in other languages, potentially introducing a selection bias and limiting the comprehensiveness of the literature analysis. (3) Potential data incompleteness and bias: Differences in data quality, coverage, and potential publication bias across platforms may lead to missing key details, resulting in gaps in the analysis.

Future directions for PROTACs in breast cancer clinical research include: (1) Overcoming intrinsic resistance: enhancing the therapeutic durability of PROTACs through multifaceted design strategies. (2) Expanding target scope: deepening exploration of key targets like HER2 and enriching the targetable protein library. (3) Broadening application scenarios: extending research to early-stage breast cancer and neoadjuvant therapy settings. (4) Covering more subtypes: expanding from ER + breast cancer to additional molecular subtypes (such as TNBC, HER2 +). (5) Exploring combination strategies: evaluating synergistic effects when combined with existing therapies such as immunotherapy. (6) Integrating AI technology: leveraging artificial intelligence to assist PROTACs design and optimization processes.

## Conclusion

This study offers an integrated analysis of bibliometric and clinical trial data, systematically mapping the developmental trajectory of PROTACs in breast cancer therapeutics. In conclusion, PROTAC technology emerges as a promising paradigm in breast cancer therapeutics, showing potential for addressing endocrine resistance in advanced ER + disease based on current trial findings. The field is rapidly evolving from foundational research into clinical reality, as evidenced by the progression of agents like ARV-471. Addressing the challenges of target diversity and translational bottlenecks will be essential to fully unlock the potential of PROTACs across the spectrum of breast cancer subtypes and treatment settings.

## Supplementary Information

Below is the link to the electronic supplementary material.

Additional file1 (DOCX 90 kb)

## Data Availability

Data is provided within the manuscript or supplementary information files.

## Abbreviations

ER: Estrogen receptor
ER +: Estrogen receptor positive
HER2: Human epidermal growth factor receptor 2
HR +: Hormone receptor positive
POI: Proteins of interest
PROTAC: Proteolysis-targeting chimera
TNBC: Triple-negative breast cancer
WoSCC: Web of Science Core Collection
