# Supplementary material for: Targeting drug resistance in breast cancer: a dual-perspective landscape analysis of PROTACs from bench to bedside
Source: Breast Cancer Res. 2026 Jun 9;28:145. doi: 10.1186/s13058-026-02320-w (PMC13480251; doi:10.1186/s13058-026-02320-w)
Supplement: Supplementary file 1 [file 13058_2026_2320_MOESM1_ESM.docx]

Table S1 The targets related to published studies

| Number | Target | Function | Publications |
| --- | --- | --- | --- |
| 1 | ER | Completely block the estrogen signaling pathway, overcoming resistance to traditional endocrine therapy. | [1-52] |
| 2 | PARP | Selectively eliminate DNA repair-deficient tumor cells, especially effective in refractory subtypes like triple-negative breast cancer. | [53-62] |
| 3 | BRD4 | Block oncogenic transcriptional programs and inhibit proliferation of therapy-resistant clones in estrogen receptor-positive breast cancer. | [63-70] |
| 4 | BET | Disrupt super-enhancer-driven oncogenic transcription networks to overcome acquired therapy resistance in estrogen receptor-positive breast cancer. | [71-76] |
| 5 | EZH2 | Eliminate repressive histone modifications to reverse immune evasion phenotypes in triple-negative breast cancer and overcome endocrine therapy resistance in estrogen receptor-positive breast cancer. | [77-81] |
| 6 | AR | Suppress tumor growth in AR+ triple-negative breast cancer and overcome enzalutamide resistance in ER+ breast cancer by blocking ligand-independent AR signaling. | [82-84] |
| 7 | p53 | Both a direct degradation target for PROTACs and a functional effector hub for MDM2-PROTACs, synergistically enhancing breast cancer cell killing. | [85-88] |
| 8 | HSP90 | Disrupting Hsp90, the central hub that stabilizes oncoproteins, can synergistically inhibit breast cancer growth and reverses drug resistance. | [89-92] |
| 9 | CDK9 | By blocking transcriptional regulation and reducing the expression of pro-survival proteins, it can induce apoptosis in breast cancer cells and hold promise for overcoming resistance to existing therapies. | [93-97] |
| 10 | CDK4/6 | By blocking cell cycle progression, it inhibits the proliferation of hormone receptor-positive breast cancer cells. | [98-104] |
| 11 | CDK12/13 | Disrupting the DNA damage repair pathway can enhance the sensitivity of breast cancer cells to PARP inhibitors, providing a novel strategy for overcoming drug resistance. | [105-107] |
| 12 | CDK1 | Blocking the mitotic process of breast cancer cells, inducing G2/M phase arrest and apoptosis, provides a novel direction for overcoming chemoresistance to drugs like paclitaxel. | [108] |
| 13 | BCL-XL | Targeted degradation of Bcl-xl, a key anti-apoptotic protein in breast cancer, overcomes resistance and selectively induces cancer cell death. | [109, 110] |
| 14 | HER2 | As a key anchor point, it can enable the targeted degradation of oncoproteins, thereby overcoming drug resistance. | [111-113] |
| 15 | STAT3 | Targeting the degradation of the oncogenic transcription factor STAT3, by eradicating its persistent activation in breast cancer, blocks oncogenic signaling, suppresses tumor growth, and overcomes drug resistance. | [114-116] |
| 16 | PI3K | By eliminating the key driver protein of the dysregulated PI3K signaling pathway in breast cancer, it can overcome resistance to traditional inhibitors and effectively suppresses tumor growth and metastasis. | [117-119] |
| 17 | ROR1 | ROR1 is an oncofetal receptor highly expressed in breast cancer but low in normal tissues. Its elimination eradicates this cancer-promoting receptor protein, blocking the signaling pathways it mediates that are critical for tumor growth, survival, and metastasis. | [120, 121] |
| 18 | FOXM1 | FOXM1, a master transcriptional regulator highly expressed in breast cancer and critical for driving cell proliferation, metastasis, and therapy resistance, represents an oncogenic hub. Eliminating this hub offers a promising strategy to curb tumor progression and overcome resistance. | [122, 123] |
| 19 | CD36 | By specifically degrading the CD36 fatty acid receptor, it can block tumor energy metabolism and metastatic pathways, thereby suppressing the invasiveness and chemoresistance of triple-negative breast cancer. | [124] |
| 20 | FGFR1/2 | By selectively degrading FGFR1/2 receptors and blocking MAPK/PI3K oncogenic signaling pathways, it can overcome trastuzumab resistance and endocrine therapy resistance in HER2-positive and luminal breast cancers. | [125, 126] |
| 21 | HDAC8 | Selective degradation of HDAC8 protein relieves transcriptional repression of tumor suppressor genes and disrupts super-enhancer networks, thereby suppressing metastatic dissemination and chemoresistance in triple-negative breast cancer. | [127, 128] |
| 22 | GPCR | Suppress breast cancer metastasis by degrading chemokine receptors to disrupt metastatic signaling and remodel the immunosuppressive tumor microenvironment. | [129] |
| 23 | RAD51 | By degrading the core DNA repair protein RAD51, it can induce synthetic lethality in HR-deficient breast cancer, while synergizing with PARP inhibitors to enhance genomic instability and overcome therapeutic resistance. | [130] |
| 24 | mTOR | Blocking the bidirectional transduction of pro-oncogenic signaling, it can overcome PI3K inhibitor resistance and suppress the activation of dormant metastatic cells in triple-negative breast cancer. | [131] |
| 25 | PDK1 | Degrading PDK1 disrupts the PI3K/AKT signaling nexus, reverses glycolytic addiction, and circumvents HER2-targeted therapy resistance in breast cancer while concurrently suppressing metastatic colonization. | [132] |
| 26 | KAT6 | Degrade the carcinogenic transcription regulator KAT6 protein, blocking its key role in driving tumor growth. | [133] |

Table S2 Clinical trial characteristics

| ClinicalTrials ID | RPOTACs | Targets | E3 ligase | Phase | | Statuses | Sponsor | Conditions | Interventions | Results availability |
| --- | --- | --- | --- | --- | --- | --- | --- | --- | --- | --- |
| NCT06206837 | ARV-471 | ER | CRBN | II | Active, not recruiting | | Pfizer | ER+/HER2- locally advanced or metastatic breast cancer | Vepdegestrant + PF-07220060 | Pending |
| NCT05501769 | ARV-471 | ER | CRBN | I | Completed | | Arvinas | ER+/HER2- locally advanced or metastatic breast cancer | Vepdegestrant + everolimus | Published/Conference |
| NCT05654623 | ARV-471 | ER | CRBN | III | Active, not recruiting | | Pfizer | ER+/HER2- locally advanced or metastatic breast cancer | Vepdegestrant vs fulvestrant | Published/Conference |
| NCT04606446 | ARV-471 | ER | CRBN | I | Recruiting | | Pfizer | ER+/HER2- locally advanced or metastatic breast cancer | Vepdegestrant + PF-07248144 + PF-07220060 | Pending |
| NCT05548127 | ARV-471 | ER | CRBN | II | Active, not recruiting | | Pfizer | ER+/HER2- locally advanced or metastatic breast cancer | Vepdegestrant + abemaciclib | Pending |
| NCT06125522 | ARV-471 | ER | CRBN | II | Active, not recruiting | | Pfizer | ER+/HER2- locally advanced or metastatic breast cancer | Vepdegestrant + samuraciclib | Pending |
| NCT05573555 | ARV-471 | ER | CRBN | II | Active, not recruiting | | Pfizer | ER+/HER2- locally advanced or metastatic breast cancer | Vepdegestrant + ribociclib | Pending |
| NCT05732428 | ARV-471 | ER | CRBN | I | Completed | | Pfizer | ER+/HER2- locally advanced or metastatic breast cancer | Vepdegestrant | Pending |
| NCT04072952 | ARV-471 | ER | CRBN | II | Active, not recruiting | | Arvinas | ER+/HER2- locally advanced or metastatic breast cancer | Vepdegestrant + palbociclib | Published/Conference |
| NCT05538312 | ARV-471 | ER | CRBN | I | Completed | | Pfizer | Healthy adults | Vepdegestrant + itraconazole | Registry posted |
| NCT05930925 | ARV-471 | ER | CRBN | I | Completed | | Pfizer | Healthy adults | Vepdegestrant | Registry posted |
| NCT05652660 | ARV-471 | ER | CRBN | I | Completed | | Pfizer | Healthy adults | Vepdegestrant + rosuvastatin | Registry posted |
| NCT05673889 | ARV-471 | ER | CRBN | I | Completed | | Pfizer | Healthy adults | Vepdegestrant + dabigatran etexilate | Registry posted |
| NCT06005688 | ARV-471 | ER | CRBN | I | Completed | | Pfizer | Healthy adults | Vepdegestrant + carbamazepine | Pending |
| NCT06275841 | ARV-471 | ER | CRBN | I | Completed | | Pfizer | Healthy adults | Vepdegestrant + esomeprazole | Pending |
| NCT07231991 | ARV-471 | ER | CRBN | I | Not yet recruiting | | Pfizer | Healthy adults | Vepdegestrant | Pending |
| NCT06645938 | ARV-471 | ER | CRBN | I | Completed | | Pfizer | Healthy adults | Vepdegestrant | Pending |
| NCT06347861 | ARV-471 | ER | CRBN | I | Completed | | Pfizer | Healthy adults | Vepdegestrant | Pending |
| NCT06256510 | ARV-471 | ER | CRBN | I | Completed | | Pfizer | Healthy adults | Vepdegestrant + midazolam | Pending |
| NCT06911788 | ARV-471 | ER | CRBN | I | Completed | | Pfizer | Healthy adults | Vepdegestrant | Pending |
| NCT05549505 | ARV-471 | ER | CRBN | II | Completed | | Arvinas | ER+/HER2- breast cancer amenable to definitive surgical resection and neoadjuvant | Vepdegestrant vs anastrozole | Pending |
| NCT05463952 | ARV-471 | ER | CRBN | I | Active, not recruiting | | Pfizer | ER+/HER2- locally advanced or metastatic breast cancer | Vepdegestrant | Registry posted |
| NCT05909397 | ARV-471 | ER | CRBN | III | Active, not recruiting | | Pfizer | ER+/HER2- locally advanced or metastatic breast cancer | Vepdegestrant + palbociclib vs letrozole + palbociclib | Pending |
| NCT01042379 | ARV-471 | ER | CRBN | II | Recruiting | | QuantumLeap Healthcare Collaborative | Invasive breast cancer | Vepdegestrant + letrozole or Vepdegestrant + abemaciclib | Published/Conference |
| NCT05080842 | AC682 | ER | VHL | I | Terminated | | Accutar Biotechnology Inc | ER+/HER2- locally advanced or metastatic breast cancer | AC682 | Pending |
| NCT05489679 | AC682 | ER | VHL | I | Terminated | | Accutar Biotechnology Inc | ER+/HER2- locally advanced or metastatic breast cancer | AC682 | Pending |
| NCT05654532 | AC699 | ER | VHL | I | Active, not recruiting | | Accutar Biotechnology Inc | ER+/HER2- locally advanced or metastatic breast cancer | AC699 | Published/Conference |
| NCT05628870 | HRS-1358 | ER | VHL | I | Recruiting | | Shandong Suncadia Medicine Co. | Metastatic or local advanced breast cancer | HRS-1358 or HRS-1358 + dalpiciclib isethionate tablets | Pending |
| NCT06555068 | HRS-1358 | ER | VHL | II | Recruiting | | Jiangsu HengRui Medicine Co. | Advanced unresectable or metastatic breast cancer | HRS-1358 + HRS-6209 | Pending |
| NCT06757335 | HP568 | ER | VHL | II | Not yet recruiting | | Hinova Pharmaceuticals Inc. | ER+/HER2- locally advanced or metastatic breast cancer | HP568 or HP568 + palbociclib | Pending |

Table S3 Main inclusion criteria and conditions of clinical trials

| ClinicalTrials ID | RPOTACs | Main inclusion criteria | Conditions |
| --- | --- | --- | --- |
| NCT06206837 | ARV-471 | The patient has undergone endocrine therapy and CDK4/6 inhibitor therapy | ER+/HER2- locally advanced or metastatic breast cancer |
| NCT05501769 | ARV-471 | The patient has undergone endocrine therapy and CDK4/6 inhibitor therapy | ER+/HER2- locally advanced or metastatic breast cancer |
| NCT05654623 | ARV-471 | The patient has undergone endocrine therapy and CDK4/6 inhibitor therapy | ER+/HER2- locally advanced or metastatic breast cancer |
| NCT04606446 | ARV-471 | The patient has undergone endocrine therapy and CDK4/6 inhibitor therapy | ER+/HER2- locally advanced or metastatic breast cancer |
| NCT05548127 | ARV-471 | The patient has undergone CDK4/6 inhibitor therapy | ER+/HER2- locally advanced or metastatic breast cancer |
| NCT06125522 | ARV-471 | The patient has undergone CDK4/6 inhibitor therapy | ER+/HER2- locally advanced or metastatic breast cancer |
| NCT05573555 | ARV-471 | The patient has undergone CDK4/6 inhibitor therapy | ER+/HER2- locally advanced or metastatic breast cancer |
| NCT05732428 | ARV-471 | The patient has undergone endocrine therapy | ER+/HER2- locally advanced or metastatic breast cancer |
| NCT04072952 | ARV-471 | The patient has undergone endocrine therapy and chemotherapy | ER+/HER2- locally advanced or metastatic breast cancer |
| NCT05538312 | ARV-471 | Healthy male and/or female participants of non-childbearing potential | Healthy adults |
| NCT05930925 | ARV-471 | Healthy male and/or female participants of non-childbearing potential | Healthy adults |
| NCT05652660 | ARV-471 | Healthy male and/or female participants of non-childbearing potential | Healthy adults |
| NCT05673889 | ARV-471 | Healthy male and/or female participants of non-childbearing potential | Healthy adults |
| NCT06005688 | ARV-471 | Healthy male and/or female participants of non-childbearing potential | Healthy adults |
| NCT06275841 | ARV-471 | Healthy male and/or female participants of non-childbearing potential | Healthy adults |
| NCT07231991 | ARV-471 | Healthy male and/or female participants of non-childbearing potential | Healthy adults |
| NCT06645938 | ARV-471 | Healthy male and/or female participants of non-childbearing potential | Healthy adults |
| NCT06347861 | ARV-471 | Healthy male and/or female participants of non-childbearing potential | Healthy adults |
| NCT06256510 | ARV-471 | Healthy male and/or female participants of non-childbearing potential | Healthy adults |
| NCT06911788 | ARV-471 | Healthy male and/or female participants of non-childbearing potential | Healthy adults |
| NCT05549505 | ARV-471 | Suitable for radical surgical resection and without bilateral ductal carcinoma in situ or invasive breast cancer | ER+/HER2- breast cancer amenable to definitive surgical resection and neoadjuvant |
| NCT05463952 | ARV-471 | The patient who is resistant to standard therapy or for which no standard therapy is available or have received | ER+/HER2- locally advanced or metastatic breast cancer |
| NCT05909397 | ARV-471 | No prior therapy | ER+/HER2- locally advanced or metastatic breast cancer |
| NCT01042379 | ARV-471 | No prior therapy | Invasive breast cancer |
| NCT05080842 | AC682 | No prior therapy | ER+/HER2- locally advanced or metastatic breast cancer |
| NCT05489679 | AC682 | The patient has undergone endocrine therapy | ER+/HER2- locally advanced or metastatic breast cancer |
| NCT05654532 | AC699 | The patient has undergone endocrine therapy and CDK4/6 inhibitor therapy | ER+/HER2- locally advanced or metastatic breast cancer |
| NCT05628870 | HRS-1358 | The patient has undergone endocrine therapy | Metastatic or local advanced breast cancer |
| NCT06555068 | HRS-1358 | No prior therapy | Advanced unresectable or metastatic breast cancer |
| NCT06757335 | HP568 | The patient has undergone endocrine therapy and chemotherapy | ER+/HER2- locally advanced or metastatic breast cancer |

**Supplementary References**

1.	Hamilton EP, Ma C, De Laurentiis M, Iwata H, Hurvitz SA, Wander SA, et al. VERITAC-2: A phase III study of vepdegestrant, a PROTAC ER degrader, versus fulvestrant in ER+/HER2- advanced breast cancer. Future Oncol. 2024 Oct 10;20(32):2447–55.

2.	Campone M, De Laurentiis M, Jhaveri K, Hu X, Ladoire S, Patsouris A, et al. Vepdegestrant, a PROTAC estrogen receptor degrader, in advanced breast cancer. N Engl J Med. 2025 Aug 7;393(6):556–68.

3.	Rej RK, Roy J, Allu SR. Therapies for the treatment of advanced/metastatic estrogen receptor-positive breast cancer: Current situation and future directions. Cancers. 2024 Jan 27;16(3):552.

4.	Wang Y, Tang SC. The race to develop oral SERDs and other novel estrogen receptor inhibitors: Recent clinical trial results and impact on treatment options. Cancer Metastasis Rev. 2022 Dec;41(4):975–90.

5.	Rej RK, Thomas JE, Acharyya RK, Rae JM, Wang S. Targeting the estrogen receptor for the treatment of breast cancer: Recent advances and challenges. J Med Chem. 2023 July 13;66(13):8339–81.

6.	Gonzalez TL, Hancock M, Sun S, Gersch CL, Larios JM, David W, et al. Targeted degradation of activating estrogen receptor α ligand-binding domain mutations in human breast cancer. Breast Cancer Res Treat. 2020 Apr;180(3):611–22.

7.	Gadd MS, Testa A, Lucas X, Chan KH, Chen W, Lamont DJ, et al. Structural basis of PROTAC cooperative recognition for selective protein degradation. Nat Chem Biol. 2017 May;13(5):514–21.

8.	Lin X, Xiang H, Luo G. Targeting estrogen receptor α for degradation with PROTACs: A promising approach to overcome endocrine resistance. Eur J Med Chem. 2020 Nov;206:112689.

9.	Choi HI, Choi J, Kim JW, Lee YH, Cho KH, Koo TS. Stability evaluation and pharmacokinetic profiling of vepdegestrant in rodents using liquid chromatography–tandem mass spectrometry. Molecules. 2024 Aug 27;29(17):4048.

10.	Toure M, Crews CM. Small‐molecule PROTACS: New approaches to protein degradation. Angew Chem Int Ed. 2016 Feb 5;55(6):1966–73.

11.	Zengerle M, Chan KH, Ciulli A. Selective small molecule induced degradation of the BET bromodomain protein BRD4. ACS Chem Biol. 2015 Aug 21;10(8):1770–7.

12.	Iwata H, Naito Y, Hattori M, Yoshimura A, Yonemori K, Aizawa M, et al. Safety and pharmacokinetics of vepdegestrant in japanese patients with ER+ advanced breast cancer: A phase 1 study. Int J Clin Oncol. 2025 Jan;30(1):72–82.

13.	Lu AS, Rouhimoghadam M, Arnatt CK, Filardo EJ, Salem AK. Proteolytic targeting chimeras with specificity for plasma membrane and intracellular estrogen receptors. Mol Pharmaceutics. 2021 Mar 1;18(3):1455–69.

14.	Burslem GM, Crews CM. Proteolysis-targeting chimeras as therapeutics and tools for biological discovery. Cell. 2020 Apr;181(1):102–14.

15.	Hamilton EP, Jeselsohn RM, Vahdat LT, Hurvitz SA. PROteolysis TArgeting chimera (PROTAC) estrogen receptor degraders for treatment of estrogen receptor–positive advanced breast cancer. Targeted Oncol. 2025 May;20(3):431–44.

16.	Zhang X, Zhang Z, Xue X, Fan T, Tan C, Liu F, et al. PROTAC degrader of estrogen receptor α targeting DNA-binding domain in breast cancer. ACS Pharmacol Transl Sci. 2022 Nov 11;5(11):1109–18.

17.	Qin H, Zhang Y, Lou Y, Pan Z, Song F, Liu Y, et al. Overview of PROTACs targeting the estrogen receptor: Achievementsfor biological and drug discovery. Curr Med Chem. 2022 July;29(22):3922–44.

18.	Gough SM, Flanagan JJ, Teh J, Andreoli M, Rousseau E, Pannone M, et al. Oral estrogen receptor PROTAC vepdegestrant (ARV-471) is highly efficacious as monotherapy and in combination with CDK4/6 or PI3K/mTOR pathway inhibitors in preclinical ER+ breast cancer models. Clin Cancer Res. 2024 Aug 15;30(16):3549–63.

19.	Shastry M, Hamilton E. Novel estrogen receptor-targeted agents for breast cancer. Curr Treat Options Oncol. 2023 July;24(7):821–44.

20.	Luo G, Li X, Lin X, Lu X, Li Z, Xiang H. Novel 11β-substituted estradiol conjugates: Transition from ERα agonizts to effective PROTAC degraders. The Journal of Steroid Biochemistry and Molecular Biology. 2022 Oct;223:106154.

21.	Pagliuca M, Donato M, D’Amato AL, Rosanova M, Russo AOM, Scafetta R, et al. New steps on an old path: Novel estrogen receptor inhibitors in breast cancer. Critical Reviews in Oncology/Hematology. 2022 Dec;180:103861.

22.	Wang L, Guillen VS, Sharma N, Flessa K, Min J, Carlson KE, et al. New class of selective estrogen receptor degraders (SERDs): Expanding the toolbox of PROTAC degrons. ACS Med Chem Lett. 2018 Aug 9;9(8):803–8.

23.	Cheng X, Hu S, Cheng K. Microneedle patch delivery of PROTACs for anti-cancer therapy. ACS Nano. 2023 June 27;17(12):11855–68.

24.	Hayhow TG, Williamson B, Lawson M, Cureton N, Braybrooke EL, Campbell A, et al. Metabolism-driven in vitro/in vivo disconnect of an oral ERɑ VHL-PROTAC. Commun Biol. 2024 May 13;7(1):563.

25.	Cyrus K, Wehenkel M, Choi E, Lee H, Swanson H, Kim K. Jostling for position: Optimizing linker location in the design of estrogen receptor‐targeting PROTACs. Chemmedchem. 2010 July 5;5(7):979–85.

26.	Naganuma M, Ohoka N, Hirano M, Watanabe D, Tsuji G, Inoue T, et al. Hydrophobic CPP/HDO conjugates: A new frontier in oligonucleotide-warheaded PROTAC delivery. RSC Med Chem. 2024;15(11):3695–703.

27.	Liang J, Wang D, Wu Y, Shi J, Xie B, Xiao R, et al. Intercepting the downstream of the estrogen receptor signaling pathway: Discovery of a potent and efficient SRC-3 PROTAC degrader for overcoming endocrine resistance breast cancer. J Med Chem. 2025 June 12;68(11):11516–42.

28.	Zhou Z, Fan H, Yu D, Shi F, Li Q, Zhang Z, et al. Glutathione-responsive PROTAC for targeted degradation of ERα in breast cancer cells. Bioorg Med Chem. 2023 Dec;96:117526.

29.	Mottamal M, Kang B, Peng X, Wang G. From pure antagonists to pure degraders of the estrogen receptor: Evolving strategies for the same target. ACS Omega. 2021 Apr 13;6(14):9334–43.

30.	Wang X, Xin L, Deng X, Dong C, Hu G, Zhou HB. Fluorescence theranostic PROTACs for real-time visualization of ERα degradation. Eur J Med Chem. 2024 Mar;267:116184.

31.	Negi A, Kesari KK, Voisin-Chiret AS. Estrogen receptor-α targeting: PROTACs, SNIPERs, peptide-PROTACs, antibody conjugated PROTACs and SNIPERs. Pharmaceutics. 2022 Nov 19;14(11):2523.

32.	Das A, Lavanya KJ, Nandini, Kaur K, Jaitak V. Effectiveness of selective estrogen receptor modulators in BreastCancer therapy: An update. Curr Med Chem. 2023 Sept;30(29):3287–314.

33.	Neerasa J, Kim B, Chung H. Efficient stereoselective synthesis of vepdegestrant (ARV‐471) via ruthenium‐catalyzed asymmetric hydrogenation. ChemistrySelect. 2025 Jan;10(4):e202405939.

34.	Yang DZ, Zhou L, Winton JA, Matschke KT, Kantaridis C, Doyle K, et al. Effect of vepdegestrant, a PROTAC oestrogen receptor degrader, on dabigatran and rosuvastatin pharmacokinetics in healthy participants. Br J Clin Pharmacol. 2025 Dec;91(12):3501–10.

35.	Xin L, Wang C, Cheng Y, Wang H, Guo X, Deng X, et al. Discovery of novel ERα and aromatase dual-targeting PROTAC degraders to overcome endocrine-resistant breast cancer. J Med Chem. 2024 June 13;67(11):8913–31.

36.	Xie B, Xu B, Xin L, Wei Y, Guo X, Dong C. Discovery of estrogen receptor α targeting caged hypoxia-responsive PROTACs with an inherent bicyclic skeleton for breast cancer treatment. Bioorg Chem. 2023 Aug;137:106590.

37.	Chen Z, Hu B, Rej RK, Wu D, Acharyya RK, Wang M, et al. Discovery of ERD-3111 as a potent and orally efficacious estrogen receptor PROTAC degrader with strong antitumor activity. J Med Chem. 2023 Sept 14;66(17):12559–85.

38.	Rej RK, Hu B, Chen Z, Acharyya RK, Wu D, Metwally H, et al. Discovery of ERD-12310A as an exceptionally potent and orally efficacious PROTAC degrader of estrogen receptor α (ERα). J Med Chem. 2024 Dec 12;67(23):20933–65.

39.	Acharyya RK, Rej RK, Hu B, Chen Z, Wu D, Lu J, et al. Discovery of ERD-1233 as a potent and orally efficacious estrogen receptor PROTAC degrader for the treatment of ER+ human breast cancer. J Med Chem. 2024 Nov 14;67(21):19010–37.

40.	Hu J, Hu B, Wang M, Xu F, Miao B, Yang CY, et al. Discovery of ERD-308 as a highly potent proteolysis targeting chimera (PROTAC) degrader of estrogen receptor (ER). J Med Chem. 2019 Feb 14;62(3):1420–42.

41.	Xie B, Yin Z, Hu Z, Lv J, Du C, Deng X, et al. Discovery of a novel class of PROTACs as potent and selective estrogen receptor α degraders to overcome endocrine-resistant breast cancer *in vitro* and *in vivo*. J Med Chem. 2023 May 25;66(10):6631–51.

42.	Jiang Y, Deng Q, Zhao H, Xie M, Chen L, Yin F, et al. Development of stabilized peptide-based PROTACs against estrogen receptor α. ACS Chem Biol. 2018 Mar 16;13(3):628–35.

43.	Peng R, Liu X, Chen CC, Guo RT, Min J. Development of PROTACs targeting estrogen receptor: An emerging technique for combating endocrine resistance. RSC Med Chem. 2025;16(3):1023–36.

44.	Dai Y, Yue N, Gong J, Liu C, Li Q, Zhou J, et al. Development of cell-permeable peptide-based PROTACs targeting estrogen receptor α. Eur J Med Chem. 2020 Feb;187:111967.

45.	Avery S, Buske JM, Chen D, Chen H, Chen X, Davidson AR, et al. Development of a commercial manufacturing process for vepdegestrant, an orally bioavailable PROTAC estrogen receptor degrader for the treatment of breast cancer. Org Process Res Dev. 2024 Nov 15;28(11):4079–90.

46.	Loren G, Espuny I, Llorente A, Donoghue C, Verdaguer X, Gomis RR, et al. Design and optimization of oestrogen receptor PROTACs based on 4-hydroxytamoxifen. Eur J Med Chem. 2022 Dec;243:114770.

47.	Haines CN, Wardell SE, McDonnell DP. Current and emerging estrogen receptor-targeted therapies for the treatment of breast cancer. Essays in Biochemistry. 2021 Nov 26;65(6):985–1001.

48.	Hu B, Hu J. Complete elimination of estrogen receptor α by PROTAC estrogen receptor α degrader ERD-148 in breast cancer cells. Breast Cancer Res Treat. 2024 Jan;203(2):383–96.

49.	Grinshpun A. Clinician’s guide to targeted estrogen receptor degradation using PROTAC in patients with estrogen receptor-positive metastatic breast cancer. Curr Opin Oncol. 2023 Nov;35(6):472–8.

50.	Disch JS, Duffy JM, Lee ECY, Gikunju D, Chan B, Levin B, et al. Bispecific estrogen receptor α degraders incorporating novel binders identified using DNA-encoded chemical library screening. J Med Chem. 2021 Apr 22;64(8):5049–66.

51.	Feng Y, Zhang Z, Zhang H, Guo H, Tan C, Xu N, et al. Aptamer proteolysis-targeting chimeras (PROTACs): A novel strategy to combat drug resistance in estrogen receptor α-positive breast cancer. ACS Pharmacol Transl Sci. 2024 Dec 13;7(12):3945–54.

52.	Zhou J, Shen R, Liu J, Deng X, Xin L, Zhou HB, et al. A novel selective estrogen receptor degrader induces cell cycle arrest in breast cancer via ERα degradation and the autophagy-lysosome pathway. Bioorg Med Chem. 2023 Mar;82:117235.

53.	Wang Y, Zhang J, Wu X, Huang L, Xiao W, Guo C. The potential of PARP inhibitors as antitumor drugs and the perspective of molecular design. J Med Chem. 2025 Jan 9;68(1):18–48.

54.	Pu C, Wang S, Luo D, Liu Y, Ma X, Zhang H, et al. Synthesis and biological evaluation of a tumor-selective degrader of PARP1. Bioorg Med Chem. 2022 Sept;69:116908.

55.	Peng X, Pan W, Jiang F, Chen W, Qi Z, Peng W, et al. Selective PARP1 inhibitors, PARP1-based dual-target inhibitors, PROTAC PARP1 degraders, and prodrugs of PARP1 inhibitors for cancer therapy. Pharmacol Res. 2022 Dec;186:106529.

56.	Pu C, Tong Y, Liu Y, Lan S, Wang S, Yan G, et al. Selective degradation of PARP2 by PROTACs via recruiting DCAF16 for triple-negative breast cancer. Eur J Med Chem. 2022 June;236:114321.

57.	Chen L, Zou Y, Sun R, Huang M, Zhu X, Tang X, et al. Minimizing DNA trapping while maintaining activity inhibition via selective PARP1 degrader. Cell Death Dis. 2024 Dec 18;15(12):898.

58.	Zhang Z, Chang X, Zhang C, Zeng S, Liang M, Ma Z, et al. Identification of probe-quality degraders for poly(ADP-ribose) polymerase-1 (PARP-1). J Enzyme Inhib Med Chem. 2020 Jan 1;35(1):1606–15.

59.	Peng X, Li Y, Qu J, Jiang L, Wu K, Liu D, et al. High affinity and low PARP-trapping benzimidazole derivatives as a potential warhead for PARP1 degraders. Eur J Med Chem. 2024 May;271:116405.

60.	Wu Y, Wu M, Zheng X, Yu H, Mao X, Jin Y, et al. Discovery of a potent and selective PARP1 degrader promoting cell cycle arrest via intercepting CDC25C-CDK1 axis for treating triple-negative breast cancer. Bioorg Chem. 2024 Jan;142:106952.

61.	Cao C, Yang J, Chen Y, Zhou P, Wang Y, Du W, et al. Discovery of SK-575 as a highly potent and efficacious proteolysis-targeting chimera degrader of PARP1 for treating cancers. J Med Chem. 2020 Oct 8;63(19):11012–33.

62.	Li G, Lin S shan, Yu Z lei, Wu X hua, Liu J wen, Tu G hui, et al. A PARP1 PROTAC as a novel strategy against PARP inhibitor resistance via promotion of ferroptosis in p53-positive breast cancer. Biochem Pharmacol. 2022 Dec;206:115329.

63.	Li Z, Ren G, Wang X, Li X, Ding L, Zhu J, et al. Tumor microenvironment responsive nano-PROTAC for BRD4 degradation enhanced cancer photo-immunotherapy. Biomaterials. 2025 Nov;322:123387.

64.	Hu J, Hu B, Xu F, Wang M, Qin C, McEachern D, et al. Precise conformational control yielding highly potent and exceptionally selective BRD4 degraders with strong antitumor activity. J Med Chem. 2023 June 22;66(12):8222–37.

65.	Chen X, Li F, Cui B, Yan Q, Qiu C, Zhu Z, et al. Liposomes-mediated enhanced antitumor effect of docetaxel with BRD4-PROTAC as synergist for breast cancer chemotherapy/immunotherapy. Int J Pharm. 2025 Jan;668:124973.

66.	Gao Y, Ni D, Li Y, Zheng J, Zhang K, Xiao Y, et al. GLUTs-facilitated targeting BRD4 degradation in breast cancer through carbohydrate-conjugated PROTACs. J Med Chem. 2025 Aug 28;68(16):17046–64.

67.	He S, Fang Y, Wu M, Zhang P, Gao F, Hu H, et al. Enhanced tumor targeting and penetration of proteolysis-targeting chimeras through iRGD peptide conjugation: A strategy for precise protein degradation in breast cancer. J Med Chem. 2023 Dec 28;66(24):16828–42.

68.	Hu J, Yang J, Zhou R, Chen K, Zhao H, Zhou Y. Discovery of a potent BRD4 PROTAC and evaluation of its bioactivity in breast cancer cell lines. Biochem Pharmacol. 2025 Nov;241:117159.

69.	Li Y, Bao K, Sun J, Ge R, Zhang Q, Zhang B, et al. Design of PROTACs utilizing the E3 ligase GID4 for targeted protein degradation. Nat Struct Mol Biol. 2025 Sept;32(9):1825–37.

70.	Kong Y, Lan T, Wang L, Gong C, Lv W, Zhang H, et al. BRD4-specific PROTAC inhibits basal-like breast cancer partially through downregulating KLF5 expression. Oncogene. 2024 Sept 23;43(39):2914–26.

71.	Noblejas-López MDM, Nieto-Jiménez C, Galán-Moya EM, Tebar-García D, Montero JC, Pandiella A, et al. MZ1 co-operates with trastuzumab in HER2 positive breast cancer. J Exp Clin Cancer Res. 2021 Dec;40(1):106.

72.	Zhou B, Hu J, Xu F, Chen Z, Bai L, Fernandez-Salas E, et al. Discovery of a small-molecule degrader of bromodomain and extra-terminal (BET) proteins with picomolar cellular potencies and capable of achieving tumor regression. J Med Chem. 2018 Jan 25;61(2):462–81.

73.	Qin C, Hu Y, Zhou B, Fernandez-Salas E, Yang CY, Liu L, et al. Discovery of QCA570 as an exceptionally potent and efficacious proteolysis targeting chimera (PROTAC) degrader of the bromodomain and extra-terminal (BET) proteins capable of inducing complete and durable tumor regression. J Med Chem. 2018 Aug 9;61(15):6685–704.

74.	Teufelsbauer M, Stickler S, Eggerstorfer MT, Hammond DC, Hamilton G. BET-directed PROTACs in triple negative breast cancer cell lines MDA-MB-231 and MDA-MB-436. Breast Cancer Res Treat. 2024 Nov;208(1):89–101.

75.	He S, Gao F, Ma J, Ma H, Dong G, Sheng C. Aptamer‐PROTAC conjugates (APCs) for tumor‐specific targeting in breast cancer. Angew Chem Int Ed. 2021 Oct 18;60(43):23299–305.

76.	Noblejas-López MDM, Nieto-Jimenez C, Burgos M, Gómez-Juárez M, Montero JC, Esparís-Ogando A, et al. Activity of BET-proteolysis targeting chimeric (PROTAC) compounds in triple negative breast cancer. J Exp Clin Cancer Res. 2019 Dec;38(1):383.

77.	Dale B, Anderson C, Park KS, Kaniskan HÜ, Ma A, Shen Y, et al. Targeting triple-negative breast cancer by a novel proteolysis targeting chimera degrader of enhancer of zeste homolog 2. ACS Pharmacol Transl Sci. 2022 July 8;5(7):491–507.

78.	Corbin J, Yu X, Jin J, Cai L, Wang GG. EZH2 PROTACs target EZH2- and FOXM1-associated oncogenic nodes, suppressing breast cancer cell growth. Oncogene. 2024 Aug 30;43(36):2722–36.

79.	Wang C, Chen X, Liu X, Lu D, Li S, Qu L, et al. Discovery of precision targeting EZH2 degraders for triple-negative breast cancer. Eur J Med Chem. 2022 Aug;238:114462.

80.	Velez J, Dale B, Park KS, Kaniskan HÜ, Yu X, Jin J. Discovery of a novel, highly potent EZH2 PROTAC degrader for targeting non-canonical oncogenic functions of EZH2. Eur J Med Chem. 2024 Mar;267:116154.

81.	Xiao B, Shi Z, Liu J, Huang Q, Shu K, Liu F, et al. Design, synthesis, and evaluation of VHL-based EZH2 degraders for breast cancer. Bioorg Chem. 2024 Feb;143:107078.

82.	Rodriguez-Gonzalez A, Cyrus K, Salcius M, Kim K, Crews CM, Deshaies RJ, et al. Targeting steroid hormone receptors for ubiquitination and degradation in breast and prostate cancer. Oncogene. 2008 Dec 4;27(57):7201–11.

83.	Wu Y, Xue J, Li J. Chemical degrader enhances the treatment of androgen receptor-positive triple-negative breast cancer. Arch Biochem Biophys. 2022 May;721:109194.

84.	Zhao L, Han X, Lu J, McEachern D, Wang S. A highly potent PROTAC androgen receptor (AR) degrader ARD-61 effectively inhibits AR-positive breast cancer cell growth in vitro and tumor growth in vivo. Neoplasia. 2020 Oct;22(10):522–32.

85.	Adams CM, Mitra R, Xiao Y, Michener P, Palazzo J, Chao A, et al. Targeted MDM2 degradation reveals a new vulnerability for p53-inactivated triple-negative breast cancer. Cancer Discovery. 2023 May 4;13(5):1210–29.

86.	Marvalim C, Datta A, Lee SC. Role of p53 in breast cancer progression: An insight into p53 targeted therapy. Theranostics. 2023;13(4):1421–42.

87.	Zhuang X, Guo Y, Sun X, Chen J, Xie S, Yang F, et al. Promising proteolysis-targeting chimera for mutant p53-R175H. ACS Omega. 2024 Nov 12;9(45):45138–46.

88.	Goerg A, Piendl G, Albert V, Ortmann O, Wege AK, Brockhoff G. Mdm2 targeting via PROteolysis TArgeting chimeras (PROTAC) is efficient in p53 wildtype, p53-mutated, and abemaciclib-resistant estrogen receptor-positive cell lines and superior to mdm2 inhibition. BMC Cancer. 2025 June 1;25(1):978.

89.	Liang X, Chen R, Wang C, Wang Y, Zhang J. Targeting HSP90 for cancer therapy: Current progress and emerging prospects. J Med Chem. 2024 Sept 26;67(18):15968–95.

90.	Jiang Q, Fu M, Tang Y, Li G, Tu G, Wu X, et al. Discovery of X10g as a selective PROTAC degrader of Hsp90α protein for treating breast cancer. Eur J Med Chem. 2023 Nov;260:115690.

91.	Liu Q, Tu G, Hu Y, Jiang Q, Liu J, Lin S, et al. Discovery of BP3 as an efficacious proteolysis targeting chimera (PROTAC) degrader of HSP90 for treating breast cancer. Eur J Med Chem. 2022 Jan;228:114013.

92.	Jiang Q, Hu Y, Liu Q, Tang Y, Wu X, Liu J, et al. Albumin-encapsulated HSP90-PROTAC BP3 nanoparticles not only retain protein degradation ability but also enhance the antitumour activity of BP3 *in vivo*. J Drug Targeting. 2023 Apr 21;31(4):411–20.

93.	Wu T, Qin Z, Tian Y, Wang J, Xu C, Li Z, et al. Recent developments in the biology and medicinal chemistry of CDK9 inhibitors: An update. J Med Chem. 2020 Nov 25;63(22):13228–57.

94.	Nie HJ, Li BF, Sun J, Yuan Y, Zhang ZG, Hu H, et al. Discovery of a potent, selective and orally bioavailable CDK9 degrader for targeting transcription regulation in triple-negative breast cancer. Bioorg Chem. 2024 Dec;153:107876.

95.	Robb CM, Contreras JI, Kour S, Taylor MA, Abid M, Sonawane YA, et al. Chemically induced degradation of CDK9 by a proteolysis targeting chimera (PROTAC). Chem Commun. 2017;53(54):7577–80.

96.	Chen W, Wu Y, Yang C, Ren W, Hou L, Liang H, et al. CDK9 targeting PROTAC L055 inhibits ERα-positive breast cancer. Biomed Pharmacother. 2024 Aug;177:116972.

97.	Noblejas-López MDM, Gandullo-Sánchez L, Galán-Moya EM, López-Rosa R, Tébar-García D, Nieto-Jiménez C, et al. Antitumoral activity of a CDK9 PROTAC compound in HER2-positive breast cancer. IJMS. 2022 May 13;23(10):5476.

98.	Steinebach C, Ng YLD, Sosič I, Lee CS, Chen S, Lindner S, et al. Systematic exploration of different E3 ubiquitin ligases: an approach towards potent and selective CDK6 degraders. Chem Sci. 2020;11(13):3474–86.

99.	Yuan K, Wang X, Dong H, Min W, Hao H, Yang P. Selective inhibition of CDK4/6: A safe and effective strategy for developing anticancer drugs. Acta Pharmaceutica Sinica B. 2021 Jan;11(1):30–54.

100.	Su S, Yang Z, Gao H, Yang H, Zhu S, An Z, et al. Potent and preferential degradation of CDK6 via proteolysis targeting chimera degraders. J Med Chem. 2019 Aug 22;62(16):7575–82.

101.	Tracy PD, Bopp E, Milner E, Garrido-Castro AC, Giordano A, Mayer EL, et al. Management of metastatic hormone receptor-positive breast cancer beyond CDK4/6 inhibitors. Curr Oncol Rep. 2025 May 28;27(7):915–33.

102.	Pu C, Liu Y, Deng R, Xu Q, Wang S, Zhang H, et al. Development of PROTAC degrader probe of CDK4/6 based on DCAF16. Bioorg Chem. 2023 Sept;138:106637.

103.	Ammazzalorso A, Agamennone M, De Filippis B, Fantacuzzi M. Development of CDK4/6 inhibitors: A five years update. Molecules. 2021 Mar 9;26(5):1488.

104.	Adon T, Shanmugarajan D, Kumar HY. CDK4/6 inhibitors: A brief overview and prospective research directions. RSC Adv. 2021;11(47):29227–46.

105.	Niu T, Li K, Jiang L, Zhou Z, Hong J, Chen X, et al. Noncovalent CDK12/13 dual inhibitors-based PROTACs degrade CDK12-cyclin K complex and induce synthetic lethality with PARP inhibitor. Eur J Med Chem. 2022 Jan;228:114012.

106.	Zhou L, Zhou K, Chang Y, Yang J, Fan B, Su Y, et al. Discovery of ZLC491 as a potent, selective, and orally bioavailable CDK12/13 PROTAC degrader. J Med Chem. 2024 Oct 24;67(20):18247–64.

107.	Yang J, Chang Y, Tien JCY, Wang Z, Zhou Y, Zhang P, et al. Discovery of a highly potent and selective dual PROTAC degrader of CDK12 and CDK13. J Med Chem. 2022 Aug 25;65(16):11066–83.

108.	Manda S, Chatakonda VK, Ugale VG, Tanwar S, Raperthi C, Borkar M, et al. Development of novel paullone-based PROTACs as anticancer agents. J Mol Struct. 2025 Apr;1327:141273.

109.	Zhang J, Zhang B, Pu C, Cui J, Huang K, Wang H, et al. Nanoliposomal bcl-xL proteolysis-targeting chimera enhances anti-cancer effects on cervical and breast cancer without on-target toxicities. Adv Compos Hybrid Mater. 2023 Apr;6(2):78.

110.	Jia Y, Han L, Ramage CL, Wang Z, Weng CC, Yang L, et al. Co-targeting BCL-XL and BCL-2 by PROTAC 753B eliminates leukemia cells and enhances efficacy of chemotherapy by targeting senescent cells. Haematologica. 2023 Apr 20;108(10):2626–38.

111.	Wang Z, Tan M, Su W, Huang W, Zhang J, Jia F, et al. Persistent degradation of HER2 protein by hybrid nanoPROTAC for programmed cell death. J Med Chem. 2023 May 11;66(9):6263–73.

112.	Hu M, Li Y, Li J, Zhou H, Liu C, Liu Z, et al. Discovery of potent and selective HER2 PROTAC degrader based tucatinib with improved efficacy against HER2 positive cancers. Eur J Med Chem. 2022 Dec;244:114775.

113.	Maneiro M, Forte N, Shchepinova MM, Kounde CS, Chudasama V, Baker JR, et al. Antibody–PROTAC conjugates enable HER2-dependent targeted protein degradation of BRD4. ACS Chem Biol. 2020 June 19;15(6):1306–12.

114.	Zhou J, Tison K, Zhou H, Bai L, Acharyya RK, McEachern D, et al. STAT5 and STAT3 balance shapes dendritic cell function and tumour immunity. Nature. 2025 July 10;643(8071):519–28.

115.	Kang Z, Li S, Li Y, Song J, Peng Y, Chen Y. Small molecular inhibitors and degraders targeting STAT3 for cancer therapy: An updated review (from 2022 to 2024). Chin Chem Lett. 2025 July;36(7):110447.

116.	Long L, Fei X, Chen L, Yao L, Lei X. Potential therapeutic targets of the JAK2/STAT3 signaling pathway in triple-negative breast cancer. Front Oncol. 2024 Apr 18;14:1381251.

117.	Zhang H, Zhang L, He Y, Jiang D, Sun J, Luo Q, et al. PI3K PROTAC overcomes the lapatinib resistance in PIK3CA-mutant HER2 positive breast cancer. Cancer Lett. 2024 Aug;598:217112.

118.	Li W, Gao C, Zhao L, Yuan Z, Chen Y, Jiang Y. Phthalimide conjugations for the degradation of oncogenic PI3K. Eur J Med Chem. 2018 May;151:237–47.

119.	Yang Z, Tong Y, Liu Y, Liu Q, Ni Z, He Y, et al. Developing selective PI3K degraders to modulate both kinase and non-kinase functions. Chin Chem Lett. 2024 Nov;35(11):109577.

120.	Li L, Huang W, Ren X, Wang Z, Ding K, Zhao L, et al. Unlocking the potential: Advancements and future horizons in ROR1-targeted cancer therapies. Sci China Life Sci. 2024 Dec;67(12):2603–16.

121.	Zhao Y, Wang H, Wu P, Zhang Y, Wang Z, Xu W. Development of nanobody-based PROTAC (NbTAC) for ROR1 degradation by RNF149. Int J Biol Macromol. 2025 Aug;319:145700.

122.	Wang K, Dai X, Yu A, Feng C, Liu K, Huang L. Peptide-based PROTAC degrader of FOXM1 suppresses cancer and decreases GLUT1 and PD-L1 expression. J Exp Clin Cancer Res. 2022 Sept 29;41(1):289.

123.	Corbin J, Yu X, Jin J, Cai L, Wang GG. EZH2 PROTACs target EZH2- and FOXM1-associated oncogenic nodes, suppressing breast cancer cell growth. Oncogene. 2024 Aug 30;43(36):2722–36.

124.	Wang Z, Pan BS, Manne RK, Chen J, Lv D, Wang M, et al. CD36-mediated endocytosis of proteolysis-targeting chimeras. Cell. 2025 June;188(12):3219-3237.e18.

125.	Uemoto Y, Lin CCA, Wang B, Ye D, Fang YV, Bikorimana E, et al. Selective degradation of FGFR1/2 overcomes antiestrogen resistance in ER+ breast cancer with FGFR1/2 alterations. Cancer Lett. 2025 June;619:217668.

126.	Wang YW, Gao YH, Wang C, Zhang PF, Wang M, Lan L, et al. Design, synthesis, and biological evaluation of novel FGFR1 PROTACs. Bioorg Chem. 2025 Feb;155:108109.

127.	Xiao Y, Hale S, Awasthee N, Meng C, Zhang X, Liu Y, et al. HDAC3 and HDAC8 PROTAC dual degrader reveals roles of histone acetylation in gene regulation. Cell Chem Biol. 2023 Nov;30(11):1421-1435.e12.

128.	Zhao C, Chen D, Suo F, Setroikromo R, Quax WJ, Dekker FJ. Discovery of highly potent HDAC8 PROTACs with anti-tumor activity. Bioorg Chem. 2023 July;136:106546.

129.	Saca VR, Huber T, Sakmar TP. G protein-coupled receptor–targeted proteolysis-targeting chimeras in cancer therapeutics. Mol Pharmacol. 2025 Feb;107(2):100013.

130.	Novel proteolysis-targeting chimera targeting RAD51 for the treatment of triple-negative breast cancer. J Physiol Pharmacol. 2025;197–207.

131.	Zhu L, Fu S, Ma L, Chen Z, Zeng Q, Li R, et al. Reversing an agonist into an inhibitor: Development of mTOR degraders. Eur J Med Chem. 2025 Sept;294:117774.

132.	Deng A, Fan R, Gou J, Sang R, Lin R, Zhao T, et al. Innovative PDK1‐degrading PROTACs transform cancer aerobic glycolysis and induce immunogenic cell death in breast cancer. Exploration. 2025 Aug;5(4):e20240031.

133.	Mukohara T, Park YH, Sommerhalder D, Yonemori K, Hamilton E, Kim SB, et al. Inhibition of lysine acetyltransferase KAT6 in ER+HER2− metastatic breast cancer: A phase 1 trial. Nat Med. 2024 Aug;30(8):2242–50.
